# JMJD5 is a human arginyl C-3 hydroxylase

**DOI:** 10.1038/s41467-018-03410-w

**Published:** 2018-03-21

**Authors:** Sarah E. Wilkins, Md. Saiful Islam, Joan M. Gannon, Suzana Markolovic, Richard J. Hopkinson, Wei Ge, Christopher J. Schofield, Rasheduzzaman Chowdhury

**Affiliations:** 1 The Department of Chemistry, University of Oxford, Mansfield Road, Oxford, OX1 3TA UK; 2 Present Address: Leicester Institute of Structural and Chemical Biology and Department of Chemistry, University of Leicester, Lancaster Road, Leicester, LE1 7RH UK; 3 Present Address: Stanford University School of Medicine, Department of Molecular and Cellular Physiology, Clark Center, Stanford, CA 94305-5345 USA

## Abstract

Oxygenase-catalysed post-translational modifications of basic protein residues, including lysyl hydroxylations and *N*^ε^-methyl lysyl demethylations, have important cellular roles. Jumonji-C (JmjC) domain-containing protein 5 (JMJD5), which genetic studies reveal is essential in animal development, is reported as a histone *N*^ε^-methyl lysine demethylase (KDM). Here we report how extensive screening with peptides based on JMJD5 interacting proteins led to the finding that JMJD5 catalyses stereoselective C-3 hydroxylation of arginine residues in sequences from human regulator of chromosome condensation domain-containing protein 1 (RCCD1) and ribosomal protein S6 (RPS6). High-resolution crystallographic analyses reveal overall fold, active site and substrate binding/product release features supporting the assignment of JMJD5 as an arginine hydroxylase rather than a KDM. The results will be useful in the development of selective oxygenase inhibitors for the treatment of cancer and genetic diseases.

## Abstract (web summary)

Jumonji-C domain containing protein 5 (JMJD5) is essential for animal development but its catalytic activity has remained elusive so far. Here the authors show that human JMJD5 is an arginyl-hydroxylase and present the cofactor, substrate and product bound JMJD5 crystal structures.

## Introduction

The Jumonji-C (JmjC) family of Fe(II) and 2-oxoglutarate (2OG) dependent oxygenases play important roles in the regulation of protein biosynthesis^1,2^. In eukaryotes, the JmjC histone demethylases (KDMs) catalyse *N*^ε^-methyl lysine- and, possibly, *N*-methyl arginine-residue demethylation of histones^3,4^. Unlike the flavin-dependent lysine-specific histone demethylases (LSDs), the JmjC KDMs catalyse the demethylation of all *N*^ε^-methyl lysine methylation states and are important regulators of transcription^5,6^. Although the effects of genetic or small-molecule intervention of the JmjC KDMs at the cellular level are often subtle, mutations to these enzymes are linked to multiple diseases, and some JmjC KDMs are current medicinal chemistry targets^7–9^.

A second group of JmjC enzymes (hydroxylases) do not have KDM activity, instead they catalyse the formation of stable alcohol products arising from the oxidation of protein residues^1^. FIH (factor-inhibiting hypoxia-inducible factor/HIF), the first JmjC enzyme to be identified, regulates the hypoxic response via C-3 hydroxylation of an Asn-residue, a post-translational modification (PTM) blocking the interaction between HIF and the CBP/p300 transcriptional coactivators^1,2^. FIH also catalyses Asn-, Asp- and His-residue hydroxylation in multiple ankyrin repeat domain (ARD) proteins^1,2^. JMJD6 catalyses Lys-residue C-5 hydroxylation of splicing regulatory proteins and, possibly, *N*,*N*-dimethyl Arg-demethylation, though the latter is controversial^10^. JMJD4 catalyses Lys-hydroxylation, but at C-4 on eukaryotic release factor 1, a modification that promotes translation termination efficiency^1,2^. The ribosomal oxygenases (RIOX) catalyse the modification of ribosomal proteins; in prokaryotes, ycfD catalyses the C-3 hydroxylation of an Arg-residue in ribosomal protein L16; its human homologues MINA53 and NO66 catalyse C-3 hydroxylation of His-residues in rpl27a and rpL8, respectively^1,2^.

JMJD5 is a JmjC domain-containing protein, present in both the nucleus and the cytoplasm^11,12^. Genetic studies reveal that deletion of JMJD5 has profound detrimental effects, including embryonic lethality^13,14^. JMJD5 has many important biological roles, including regulation of cell cycle and pluripotency (via regulating cyclin-dependent kinase inhibitor 1 (CDKN1A) levels^15,16^), p53 signalling and mitosis (via association with spindle microtubules^17,18^), glucose metabolism and chromosome stability (via interactions with pyruvate kinase M2/PKM2^19^ and RCCD1^20^), and osteoclast differentiation (by destabilising nuclear factor of activated T-cells, cytoplasmic 1 (NFATC1)^21^). JMJD5 is identified being as pro-oncogenic, in colon^22^ and breast^23^ cancer.

Despite its important biological roles, the biochemical function of JMJD5 has been elusive. Early studies identified JMJD5 as a histone H3K36me_2_ demethylase regulating cyclin A1 expression^11^; however, this assignment has not been validated subsequently^21,24,25^. Recently, evidence has been reported that JMJD5 has aminopeptidase activity on histone tails^26,27^. Thus, JMJD5 is one of the few human JmjC oxygenases for which a clear biochemical function has not been defined. Given the evidence for the biological importance of JMJD5, which is unusually clear compared to most other JmjC proteins, and that other (related) JmjC enzymes are being pursued as pharmaceutical targets, it is important to assign the catalytic role of JMJD5. Here we report evidence that JMJD5 is a JmjC arginyl-hydroxylase, the first such catalytic activity identified for a human enzyme.

## Results

### JMJD5 catalyses C-3 arginyl hydroxylation

**Fig. 1:**
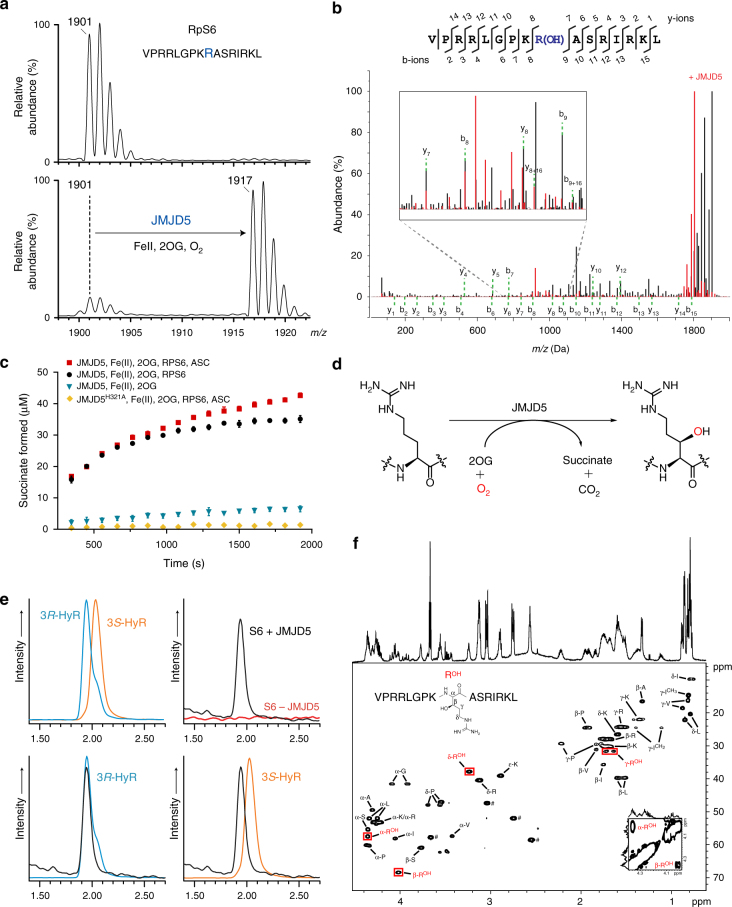
JMJD5 catalyses stereoselective C-3 hydroxylation of Arg137 of the 40S ribosomal protein S6 (RPS6). **a** MALDI-MS spectra for RPS6_129–144_ showing the +16 *m/z* shift following treatment with JMJD5 and cofactors. **b** MS/MS analysis of the modified RPS6_129–144_ indicating a +16 shift on y and b ion fragments y_8_–y_14_/b_9_–b_15_, which is not observed for ion fragments y_1_–y_7_/b_2_–b_8_, implying hydroxylation at RPS6 R137; observed/predicted masses for the fragment ions are given in Supplementary Fig. 5. **c** Plot of succinate formation monitored by ^1^H-NMR. Succinate formation from a reaction with 10 μM JMJD5, 50 μM RPS6, 200 μM 2OG and 100 μM Fe(II) (black) is compared to that without RPS6 peptide (blue), with ascorbate (red), and with an inactive H321A variant substituting for wild type (yellow). Results are the mean ± s.e.m. (*n* = 3). **d** JMJD5-catalysed arginyl hydroxylation. **e** Extracted ion chromatograms (*m/z* = 345) from LC-MS analysis of synthetic (3*R*)- and (3*S*)-hydroxyarginine (HyR) standards and amino-acid hydrolysates from unmodified/hydroxylated RPS6_129–144_. Peaks at 1.93 and 2.02 min correspond to (3*R*)- and (3*S*)-hydroxyarginine, respectively. **f**^1^H–^13^C HSQC spectra of hydroxylated RPS6_129–144_ (VPRRLGPKR^OH^ASRIRKL), labelled with aa assignments, supporting R137 C-3 hydroxylation. Red boxes highlight the hydroxyarginine (R^OH^). Inset bottom right: ^1^H–^1^H COSY spectrum showing the correlation between C-2-R^OH^ and C-3-R^OH^ protons. ♯ Indicates residual HEPES buffer

We began by analysing whether JMJD5 can convert 2OG into succinate without substrate, as observed for many 2OG-oxygenases. By ^1^H-NMR, we observed low levels of JMJD5/Fe(II)-dependent 2OG to succinate conversion, which was inhibited by the broad-spectrum 2OG-oxygenase inhibitor *N*-oxalylglycine (NOG) (Fig. 1). Substitution of one of the Fe(II)-binding residues (H321, as crystallographically observed in JMJD5 structures without substrate)^24,25^ with alanine significantly reduced 2OG turnover (Fig. 1). These results support the assignment of JMJD5 as a functional 2OG-oxygenase.

Bioinformatic and structural analyses suggest JMJD5 is more closely related to the JmjC-hydroxylases than JmjC KDMs (Supplementary Fig. 1)^1,24,25^. However, because JMJD5 has been assigned as a KDM^11^, we investigated both hydroxylase and KDM activities for recombinant JMJD5 using reported substrate(s) and binding proteins—H3K36me_2_ (for demethylation activity)^11,28^ and p53^17^, NFATC1^21^, PKM2^19^ and RCCD1^20^ (for hydroxylation activity).

To investigate KDM activity for JMJD5, we employed a mass spectrometry (MS)-based assay, using conditions enabling demethylation of histone *N*^ε^-methylated lysine residues by other KDMs^29^. We tested synthetic histone H3 fragment peptides (21-mers) containing all three methylation states (Kme_3_/Kme_2_/Kme_1_) at K4, K9, K27 and K36 sites, but observed no evidence for demethylation (or other modifications), including with the reported H3K36me_2_ substrate^11^ (Supplementary Fig. 2). In addition to wild type, an N-terminally truncated JMJD5 (aa 183–416) was tested because the JMJD5 N-terminus (aa 1–182) is reported to constitute an 'inhibitory domain' that might block its demethylase activity^24^. The truncated JMJD5, however, also did not manifest KDM activity.

We therefore investigated whether JMJD5 has any hydroxylase activity by synthesising peptides spanning all residues in the reported JMJD5 interacting proteins (p53^17,18^, NFATC1^21^, PKM2^19^ and RCCD1^20^) and testing them using MS assays. The results reveal a JMJD5-dependent shift of +16 Da on a single peptide comprising residues 134–150 of human RCCD1, which was abrogated by alanine substitution of the arginine at position 141 in RCCD1 (Supplementary Fig. 3 and Supplementary Table 1). This observation suggested the possibility of JMJD5-catalysed arginyl-hydroxylation, which is notable, because to date there has been no report of an arginyl-hydroxylase in humans, or indeed eukaryotes. This finding motivated the investigations of further JMJD5-catalysed hydroxylations.

### JMJD5 catalyses RPS6 arginyl hydroxylation

Recent work has revealed roles for structurally related JmjC-hydroxylases in regulating protein synthesis, via post-translational hydroxylations of ribosomal and associated proteins^1,2^. We therefore considered that JMJD5 might catalyse hydroxylation of proteins involved in translation. We screened ∼1000 peptides derived from human ribosomal proteins (Supplementary Data 1) using an MS-based assay, and identified a peptide derived from the 40S ribosomal protein S6 (RPS6) that showed a mass shift of +16 Da following incubation with JMJD5, Fe(II), 2OG and ascorbate, consistent with a single hydroxylation (Fig. 1). This shift was not observed in the absence of supplemented Fe(II) or 2OG, and was inhibited by addition of NOG (Supplementary Fig. 4).

Alanine scanning and tandem mass spectrometric (MS/MS) analysis of the modified peptide revealed that the hydroxylated residue is likely an arginine, corresponding to R137 in human RPS6 (Fig. 1, Supplementary Fig. 5 and Supplementary Table 3). The activity of JMJD5 is apparently specific to arginine residues, as substitution of the target arginine with other residues, including lysine, did not enable hydroxylation (Supplementary Fig. 6). JMJD5 did not catalyse hydroxylation/demethylation of an *N*^ε^-dimethyl lysine residue at this position, nor was it able to catalyse any modification of mono- or di-methyl arginine residues (in the context of the RPS6 peptide).

Nuclear magnetic resonance (NMR) spectroscopy and amino-acid analysis of the modified RPS6 peptide confirmed the presence of hydroxyarginine and revealed that JMJD5 catalyses stereoselective formation of the (2*S*,3*R*)*-*arginine stereoisomer (Fig. 1). The same stereoisomer is produced by the bacterial arginyl-hydroxylase ycfD on its L16 substrate^30,31^. However, JMJD5 and ycfD do not exhibit any cross-reactivity on their respective ribosomal protein substrates (Supplementary Fig. 7).

We further investigated the substrate preference of JMJD5 by incubating equimolar amounts of RCCD1 and RPS6 peptides. The results reveal that, within the context of peptide substrates, JMJD5 displays a clear preference for hydroxylation of RPS6 over RCCD1 (Supplementary Fig. 8). Kinetic analyses reveal that JMJD5 catalyses RPS6 fragment Arg137 hydroxylation with a several fold lower *K*_m_ value than for RCCD1 (60.4 µM and >300 µM, respectively) (Supplementary Fig. 8).

### Structures imply JMJD5 is an arginyl hydroxylase

Although the animal JmjC KDMs and hydroxylases likely have common evolutionary origins in prokaryotic oxygenases, structural studies have revealed emerging fold and active site features characteristic of the two subfamilies^30,32^. Although structures of JMJD5 in the absence of substrate are reported^24,25^, these do not inform on the relationship of the Fe-linked reactive intermediate in 2OG-oxygenase catalysis with the substrate; we therefore initiated crystallographic studies on JMJD5 substrate complexes with an aim of investigating the structural basis for its catalytic activity.

**Fig. 2:**
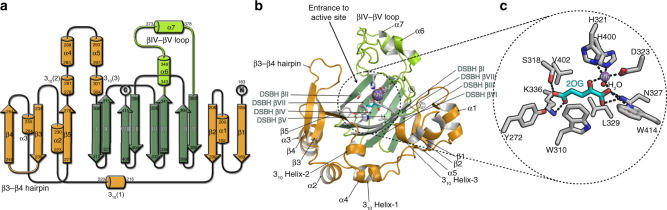
Overview of the JMJD5 structure. Topology (**a**) and ribbon representation (**b**) of the JMJD5.Mn.2OG complex and the active site close-up (**c**) (PDB: 6F4S). DSBH secondary structure elements are labelled I–VIII (green). The JMJD5 fold contains 7 α-helices, 13 β-strands and 3 3_10_ helices. The N-terminal domain preceding the DSBH is in orange and the remainder of the structure, including the DSBH βIV–V insert, in light green. 2OG is shown by cyan sticks and the metal by a purple sphere

In attempts to crystallise JMJD5, including full-length and N-terminally truncated (aa 153–416 and aa 183–416) constructs, we obtained diffraction quality crystals with JMJD5_153-416_ and JMJD5_183-416_, with the latter diffracting to substantially higher resolution (1.1–1.7 Å). We obtained multiple JMJD5 structures in complex with 2OG or the broad-spectrum inhibitor NOG (IC_50_ 6 ± 1.4 µM for JMJD5) in the *P*3_2_21 (JMJD5_153-416_) or *P*2_1_2_1_2_1_ (JMJD5_183-416_) space groups. Consistent with previous reports^24,25^, JMJD5 residues 174–416 fold into mixed helical and β-strand topology comprising seven α-helices, 13 β-strands and 3 3_10_ helices (Fig. 2a,b). The core architecture is a distorted double-stranded β-helix (DSBH) fold, an asymmetric barrel-like structure formed by eight antiparallel β-strands in major (I, VIII, III, VI) and minor (II, VII, IV, V) sheets, which is archetypical of 2OG-oxygenases^33^ (DSBH β-strands are in Roman numerals). Several α- and 3_10_-helices located N-terminal to the DSBH support the core structure. The active site is located at the relatively open end of the DSBH barrel; the other end is more 'closed' by the N-terminal β1 (aa 183–188) (Fig. 2a,b).

Like most 2OG-oxygenases, the JMJD5 active site contains a metal centre, which is octahedrally coordinated by an HXD..H motif (H321 and D323/βII, H400/βVII), the 2OG oxalyl group and a water molecule, which is likely replaced by a dioxygen during catalysis. The 2OG C1-carboxylate also makes hydrogen bonding interactions with the sidechain –NH_2_ of N327 (βIII) (Fig. 2c). Like FIH and other JmjC-hydroxylases^33^, the 2OG C5-carboxylate is positioned to form electrostatic interactions with a lysine (K336) from DSBH βIV and a tyrosine (Y272) from β5, which is located immediately N-terminal to DSBH βI and which forms an N-terminal extension to the DSBH major sheet (Fig. 2). A distinctive feature of 2OG-binding by JMJD5, compared to most other 2OG-oxygenases^33^, is that the 2OG C5-carboxylate interacts with a third polar residue, a serine (S318 from βII). The two methylenes of 2OG are tightly bound in a hydrophobic region formed by the sidechains of W310 (βI), L329 (βIII), and V402 (βVII) (Fig. 2c). Substitution of H321 or W310 by Ala led to loss of activity (Supplementary Fig. 9). The multiple interactions between the 2OG and JMJD5 likely reflect tight co-substrate binding and are consistent with the relatively low *K*_m_ for 2OG (9.5 µM) (Supplementary Fig. 8d) and the low level of JMJD5-catalysed substrate-uncoupled turnover of 2OG (Fig. 1).

**Fig. 3:**
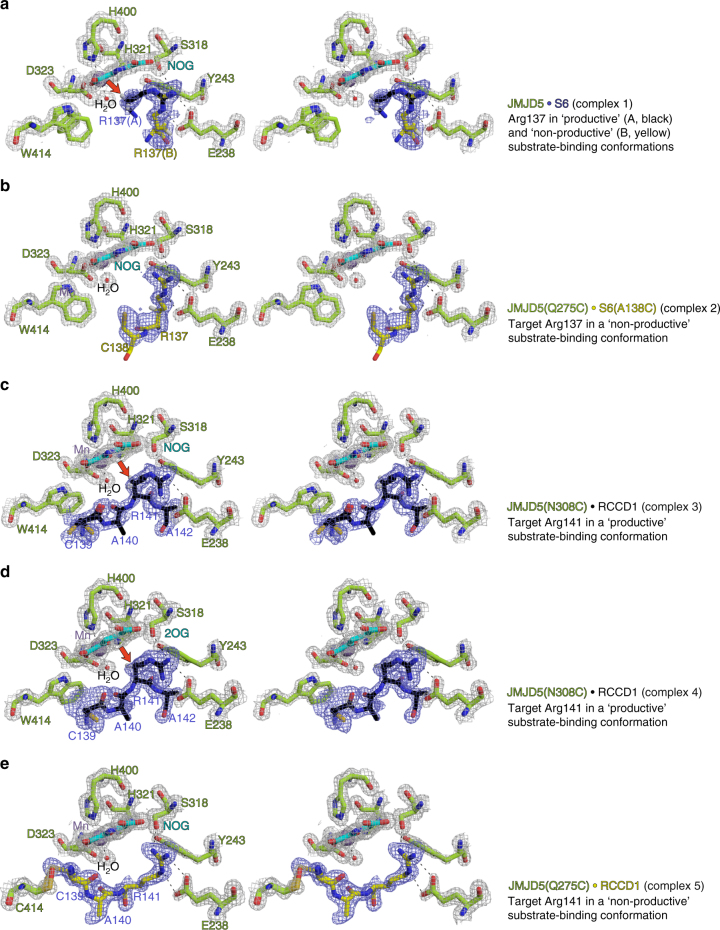
Stereoviews of JMJD5.substrate complexes showing likely productive and non-productive arginine-binding modes. **a** JMJD5.Mn.NOG.RPS6 (Complex 1, PDB: 6F4P), **b** JMJD5(Q275C).Mn.NOG.RPS6(A138C) (Complex 2, PDB: 6F4Q), **c** JMJD5(N308C).Mn.NOG.RCCD1 (Complex 3, PDB: 6F4R), **d** JMJD5(N308C).Mn.2OG.RCCD1 (Complex 4, PDB: 6F4S) and **e** JMJD5(W414C).Mn.NOG.RCCD1 (Complex 5, PDB: 6F4T). The target arginine in both RPS6 (R137) and RCCD1 (R141) binds in a shallow channel on the JMJD5 surface forming hydrophobic interactions with Y243/W310 and hydrogen bonds with E238/ S318 in both 'productive' (Complex 1/A, Complex 3 and Complex 4) and 'non-productive' (Complex 1/B, Complex 2 and Complex 5) conformations. 2*F*_o_−*F*_c_ (grey meshes) for JMJD5 and difference electron density (*F*_o_−*F*_c_omit map, blue) for substrates are contoured to 1.2−1.5σ and 3σ, respectively. Red arrows indicate hydroxylated C–H bonds

Initial attempts to obtain JMJD5.substrate (with both RPS6 and RCCD1) complex structures yielded a crystal structure of JMJD5.RPS6 complex (Complex 1) in which difference electron density (*F*_o_–*F*_c_) was only observed for the RPS6 target arginine near the active site in two side-chain conformations (A and B, Fig. 3a). In both conformations (A and B), the nearly overlapped RPS6 argininyl-guanidino group is apparently tightly bound via a salt bridge with E238 (which is the only acidic residue within 12 Å of the active site metal) and a cation–π interaction with Y243, which is located on the β3–β4 loop N-terminal to DSBH (Fig. 3a). In conformation A, these interactions project the target argininyl C-3 hydrogens towards the metal (metal–β-CH_2_, 4.1 Å). By contrast, in conformation B, the argininyl C-3 is ~9.0 Å away from the metal centre indicating that in conformation B, the residue is not positioned in a catalytically productive manner (Fig. 3a). Consistent with their crystallographically implied importance in substrate binding, the E238A and Y243A variants were inactive under standard assay conditions (Supplementary Fig. 9).

**Fig. 4:**
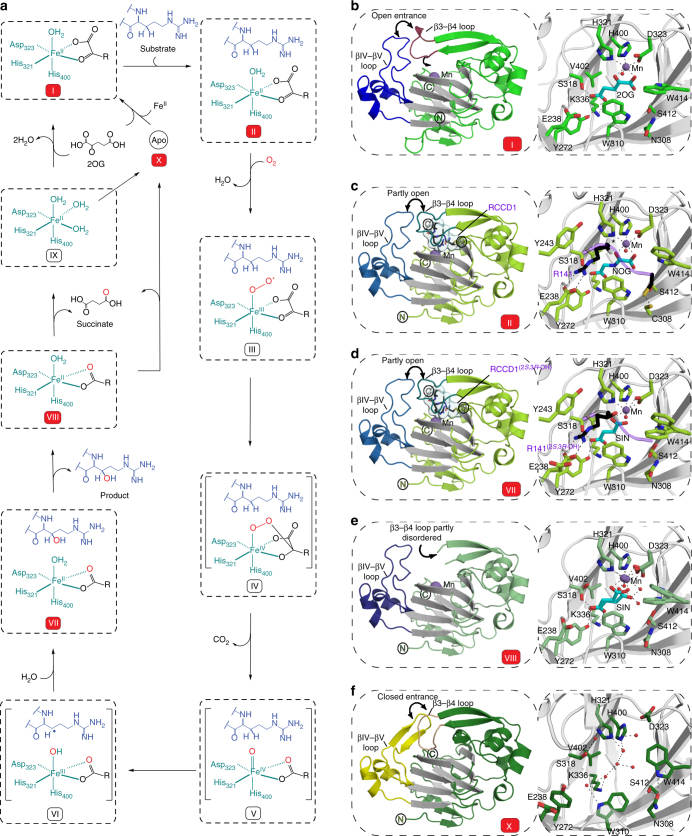
Conformational changes observed in complexes representing steps in JMJD5 catalysis. **a** Proposed mechanism of JMJD5-catalysed (3*R*)-Arg-hydroxylation (reactive intermediates in parentheses), **b** JMJD5.Mn.2OG, **c** JMJD5.Mn.NOG.RCCD1(139-142) (Complex 3), **d** JMJD5.Mn.succinate.RCCD1^(2*S*,3*R*-OH)^ (product) modelled complex, **e** JMJD5.Mn.succinate and **f** apo-JMJD5. The figure illustrates global and local changes occurring on substrate/cosubstrate binding and product release, which is a characteristic feature of JMJD5 catalysis. Two mobile regions are involved in JMJD5 catalysis: (i) the β3–β4 loop (N-terminal to the DSBH, aa 234–254, raspberry to cream), and (ii) the DSBH βIV–βV loop (aa 342–381, different blue shades). Analogous loops are often involved in substrate binding by other JmjC-hydroxylases and KDMs, including FIH^37^, RIOX^30^, KDM4A^57,58^ and PHF8^59^. The β3-β4 loop, which is often relatively short as in some KDMs, is 'open' in the JMJD5.Mn.2OG-, 'partly open' in the substrate-, and disordered in the succinate-complexes. In all JMJD5 structures, except for apo-JMJD5, the βIV–βV loop adopts a similar conformation (i.e., partly helical); however, in the apo structure, part of βIV–βV loop (aa 351–361) adopts a hairpin conformation. Note the conformational changes in the sidechains of active site residues involved in catalysis. The JMJD5.Mn.succinate.RCCD1^(2*S*,3*R*-OH)^ complex was modelled using the JMJD5(N308C).RCCD1 (Complex 3) and JMJD5.succinate structures as templates

The lack of observed difference density (*F*_o_−*F*_c_) for the RPS6 peptides, excepting RPS6-R137, suggests that JMJD5 likely forms an unstable/less stable complex with the RPS6 fragment, possibly reflecting the relatively high *K*_m_ value for RPS6 (60.4 μM, Supplementary Fig. 8e). We therefore pursued a site-specific 'disulphide enzyme-substrate cross-linking' strategy (Supplementary Fig. 10)^30,34–36^, which enabled us to obtain four structures of JMJD5(Q275C).Mn.NOG.RPS6(A138C), JMJD5(N308C).Mn.NOG.RCCD1, JMJD5(N308C).Mn.2OG.RCCD1 and JMJD5(W414C).Mn.NOG.RCCD1 as cross-linked complexes (Complexes 2–5, respectively). In these structures, clear electron density was observed for the substrate backbones that enabled modelling of residues other than the target arginine (RPS6 aa 137–138 and RCCD1 aa 139–142, Fig. 3b–e). Compared to Complex 1, single sidechain conformations for the target arginine residues corresponding to 'productive' (A in Complex 1) and 'non-productive' (B in Complex 1) orientations were observed in Complexes 3/4 and 2/5, respectively (Fig. 3). Comparisons of the substrate complexes with those containing 2OG and of apo-JMJD5 imply 'induced fit' on substrate binding involving substantial conformational changes in two loops and multiple active site residues (see below and Fig. 4).

We were unable to obtain a JMJD5 structure complexed with the hydroxylated product, but did obtain a JMJD5.Mn.succinate (decarboxylation product) complex structure; this structure manifests substantial conformational changes relative to the 2OG/NOG complexes (with/without substrate) including in DSBH residues, such as the Fe(II) binding residue D323 (Fig. 4e and Supplementary Fig. 11).

**Fig. 5:**
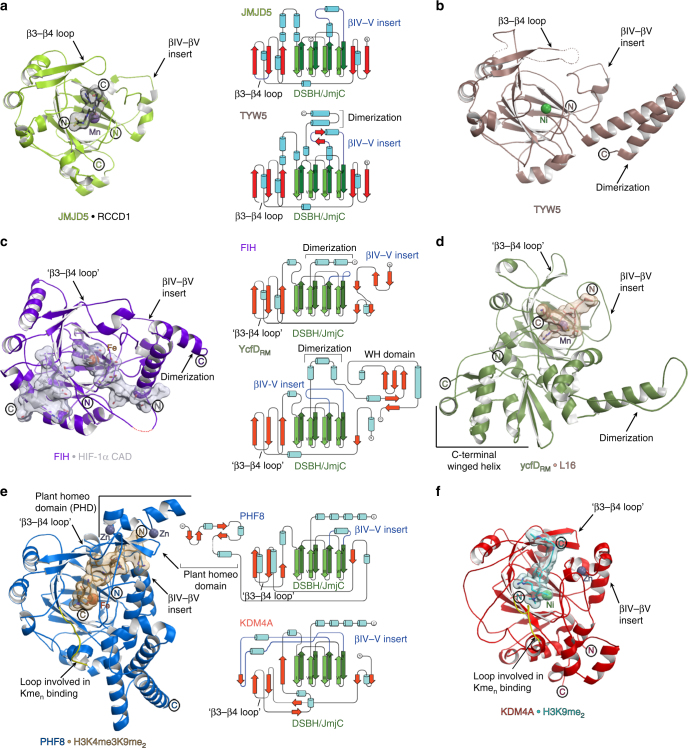
Relationship of JMJD5 structure/topology with those of homologous JmjC-hydroxylases and JmjC KDMs. **a**–**f** Ribbon representations of JMJD5.Mn.NOG.RCCD1(139–142) (PDB: 6F4R) (**a**), TYW5.Ni (PDB: 3AL5) (**b**), FIH.Fe.NOG.HIF-1α (786–826) (PDB: 1H2K) (**c**), ycfD_RM_.Mn.NOG.L16(72–91) (PDB: 4CUG) (**d**), PHF8.Fe.NOG.H3K4me_3_K9me_2_(2–25) (PDB: 3KV4) (**e**) and KDM4A.Ni.NOG.H3K9me_2_(7–14) (PDB: 2OX0) (**f**). Middle panels show topologies as indicated, with DSBH core elements (βI–βVIII) in green, helices in cyan, additional β-strands in red, random coils in black, and the insert between the fourth and fifth DSBH β-strands in blue. JMJD5 catalysis involves substantial conformational changes in the β3–β4 and βIV–V loop regions (loops analogous to JMJD5 β3–β4 loop are labelled 'β3–β4 loop' in other JmjC-oxygenases); such movements have not been observed around the JmjC KDM active sites on *N*^ε^-methylated lysine binding. In addition, JMJD5 lacks (i) the extended flexible loop region, immediately N-terminal to βI (yellow), which is involved in binding *N*^ε^-methylated lysines (Kme_n_) and (ii) the chromatin- and Zn-binding domains present in most JmjC KDMs (note, structures of PHF8 and KDM4A are only of catalytic domains). Thus, the overall JMJD5 fold together with its unique substrate binding features supports its assignment as a JmjC-hydroxylase and not a KDM

The combined structural analyses identify mobile regions involved in substrate binding/product release during JMJD5 catalysis, i.e., the β3–β4 loop, N-terminal to DSBH (aa 234–254), and the DSBH βIV–βV loop (aa 342–381, Fig. 4b–f). Except in the apo-JMJD5 structure, the βIV–βV loop, which is involved in substrate binding in other JmjC-hydroxylases including FIH^37^ and ycfDs^30^ (Fig. 5), adopts a partial helical conformation in all JMJD5 structures (Fig. 4b–e). However, in the apo-JMJD5 structure, part of the βIV–βV loop (aa 351–361) adopts a hairpin conformation (Fig. 4f). By contrast, the conformations of the β3–β4 loop (aa 241–248) vary in all JMJD5 complexes (Fig. 4b–f). In the 2OG complex structures, part of β3–β4 loop is either disordered^24^, or has an 'open' conformation relative to the substrate bound form. In the substrate complexes, the β3–β4 loop folds to enclose the target arginine region and active site. In the succinate complex, part of the β3–β4 loop (aa 241–248) is disordered, whereas in the apo-JMJD5 structure the β3–β4 loop, together with βIV–βV loop, encloses the active site entirely (Fig. 4). Thus, the combined structural analyses reveal that the β3–β4 and βIV–βV loops likely have a 'gating' role to facilitate substrate binding and product release during catalysis.

## Discussion

The finding that the transcription machinery is extensively regulated by 2OG-oxygenases, including the HIF hydroxylases and JmjC KDMs, raised the question as to whether they regulate translation^1,2^. It is now clear that some 2OG-oxygenases (e.g., the RIOX and RPS23 prolyl hydroxylases) catalyse PTMs to ribosomal (and associated regulatory) proteins and tRNA^1,2^. However, only in a few cases have the 2OG-oxygenases been shown to be crucial in animal biology; JMJD5 is one such enzyme and is involved in the regulation of cell growth/division processes that are inherently linked to translational capacity^14,15,17,38^.

**Fig. 6:**
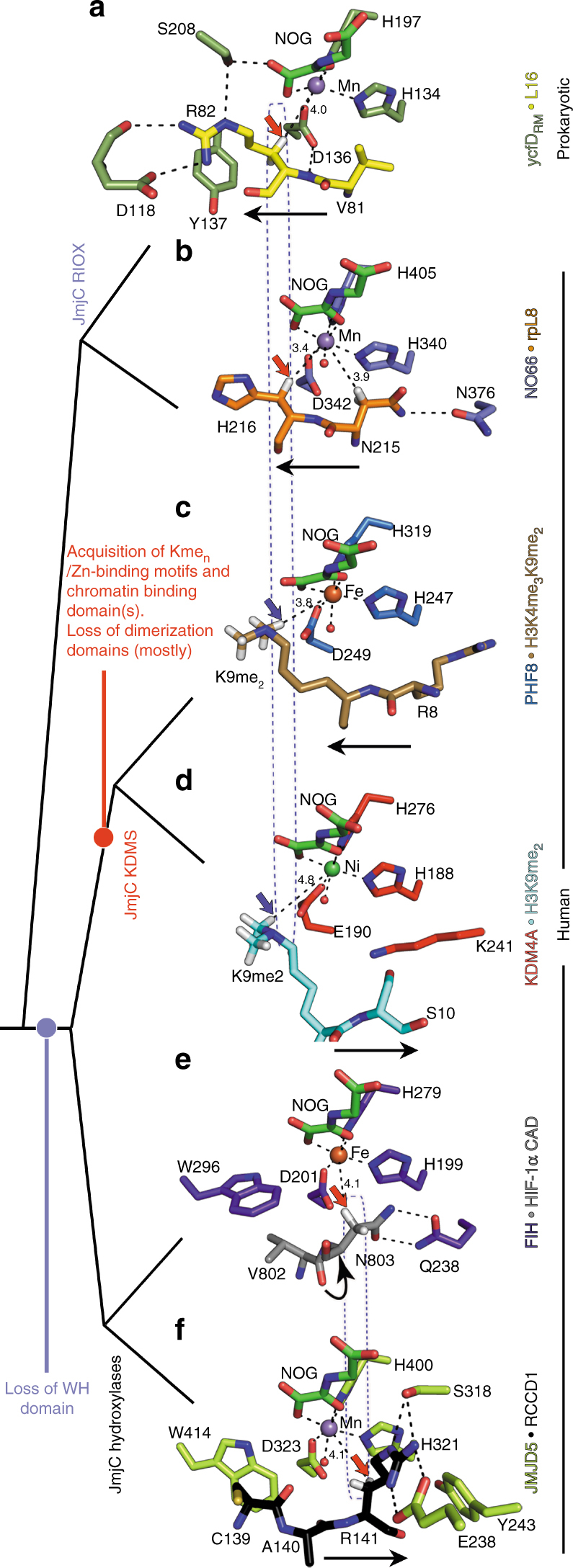
Comparison of the active site substrate binding modes for JMJD5 and related JmjC enzymes. Red/blue arrows indicate hydroxylation/demethylation sites, respectively. Active-site metals (Fe or Fe surrogates, Mn/Ni) are colour-coded spheres. Values represent distances (Å) from the metal to the oxidised carbon. **a** ycfD_RM_.Mn.NOG.L16(72–91) (PDB: 4CUG), **b** NO66.Mn.NOG.RPL8(205–224) (PDB: 4CCO), **c** PHF8.Fe.NOG.H3K4me_3_K9me_2_(2–25) (PDB: 3KV4), **d** KDM4A.Ni.NOG.H3K9me_2_(7–14) (PDB: 2OX0), **e** FIH.Fe.NOG.HIF-1α (786–826) (PDB: 1H2K), **f** JMJD5.Mn.NOG.RCCD1(139–142) (PDB: 6F4R). Note variations in the *N*- to *C*-substrate directionality in the active site cleft (indicated by black arrows), and variations in the hydroxylation site relative to the metal (boxed). JMJD5 binds substrates (**f**) with the same substrate *N*/*C-*directionality, as for most KDMs (KDM4A, KDM6B and KDM6A, **d**), but differing from that for RIOX (**a**, **b**) and for one KDM, PHF8 (and likely other KDM2/7 subfamily members, **c**). Overall, the mode of JMJD5-catalysed hydroxylation more closely resembles that of FIH (**e**) compared to other JmjC-hydroxylases and KDMs, consistent with the proposal that the FIH/JMJD5 subgroup of JmjC-hydroxylases evolved from prokaryotic RIOX including by loss of the 'Winged helix' (WH) domains, which are located at the C-terminus of all RIOX^30,32^

Building from an extensive peptide-based screen for potential JMJD5 substrates, our results provide evidence that JMJD5 is a JmjC-hydroxylase, catalysing the stereoselective C-3 hydroxylation of arginyl residues in sequences from proteins involved in chromatin stability (RCCD1) and translation (RPS6). In support of this assignment of the catalytic role of JMJD5, comparisons of JMJD5 structures with those of other 2OG-oxygenases reveal that the overall JMJD5 fold is more similar to the JmjC-hydroxylases than the KDMs, as is its substrate binding both in terms of the relationship of the substrate arginyl-residue relative to the active site Fe(II) and more general features (Figs. 5 and 6).

The most similar human JmjC-hydroxylases to JMJD5 are (in order of decreasing similarity): the tRNA hydroxylase TYW5 (PDB: 3AL5^39^, Cα RMSD = 1.9, Z = 26.2), JMJD7 (PDB: 4QU2, Cα RMSD = 1.9, Z = 25.3;), the asparaginyl hydroxylase FIH (PDB: 1H2K^37^, Cα RMSD = 2.2, Z = 24.4), and the lysyl hydroxylase JMJD6 (PDB: 3K2O^40^, Cα RMSD = 2.8, Z = 21.3). JMJD5 is more distantly related, at least in terms of overall structure, to the prokaryotic arginyl-hydroxylases, ycfD_EC_ (PDB: 4LIT^30^, Cα RMSD = 3.1, Z = 15.9) and ycfD_RM_ (PDB: 4CUG^30^, Cα RMSD = 3.5, Z = 15.7) (from *Escherichiacoli* and *Rhodothermus marinus*, respectively), with which it shares a common biochemical function, i.e., (2*S*,3*R*)-arginyl-hydroxylation, and which likely reflect evolutionary precursors of JMJD5 and other animal JmjC enzymes^30,32^.

A unique feature of substrate binding at the JMJD5 active site among the JmjC enzymes analysed concerns the crystallographic observation of two conformations for the substrate arginine sidechain—one apparently productive and one not (Fig. 3). The productive conformation was observed for both RPS6 (Complex 1/A) and RCCD1 (Complexes 3 and 4) and is analogous to other JmjC enzyme-substrate complexes, notably for FIH^37^ and ycfD_RM_^30^. Interestingly, although JMJD5 and ycfD_RM_ bind their respective substrates with different *N*/*C*-directionality through the active site channel, the general binding mode of the target Arg is similar in the productive conformation. For both the bacterial and human arginyl hydroxylases, the target arginine residues are bound in hydrophobic pockets defined by Y243_JMJD5_ and W310_JMJD5_ (Y137_ycfD_ and M120_ycfD_) and are positioned to form hydrogen-bonds to E238_JMJD5_ and S318_JMJD5_ (D118_ycfD_ and S208_ycfD_) (Figs. 6a, f). The non-productive conformation as observed for both RPS6 and RCCD1 in JMJD5 Complexes 1/B, 2 and 5 (but not in ycfD or FIH) may reflect a substrate entry/product release route, though a role in binding one of the other arginine residues present in the substrate sequence at some stage in catalysis cannot be ruled out.

Comparison of JMJD5 with the available substrate structures for other JmjC-hydroxylases (i.e., FIH and ycfD_RM_/RIOX) is interesting from catalytic and evolutionary perspectives (Fig. 6). Although JMJD5 and FIH catalyse C-3 hydroxylations of different residues with different stereoselectivities (i.e., 2*S*,3*R* vs. 2*S*,3*S*), the positioning of hydroxylated methylenes relative to the metal is strikingly similar, i.e., they nearly superimpose in overlaid structures (Fig. 6e and 6f), demonstrating that the reactive oxidising intermediates (Fe^IV^ = O) react from similar coordination positions in both FIH and JMJD5. By contrast, although JMJD5 and prokaryotic ycfD both catalyse C-3 hydroxylations of arginine residues with the same stereoselectivity (i.e., 2*S*, 3*R*), the positioning of hydroxylated methylenes differs substantially (Fig. 6a, f). Thus, the observed mode of JMJD5 hydroxylation likely evolved away from that of the RIOX, both by altering the coordination position from which the ferryl-oxo reacts and by altering the directionality of substrate binding through the active site.

Recent studies have proposed aminopeptidase activity for JMJD5 on histone tails containing methylated arginines^26^ or *N*^ε^-monomethyl-lysines^27^. While all of our 'productive' complexes (1/A, 3 and 4) are consistent with those reported for other JmjC-hydroxylases in terms of the relationship between the active site metal and the oxidised C–H bond^41,42^, neither the 'productive' nor the 'non-productive' complex structures (Fig. 3) appear to be consistent with stereoselectively favoured efficient metal-based backbone hydrolysis reactions. Thus, although there is precedent for 2OG -oxygenases being part of multi-function proteins and for the presence of the DSBH in hydrolytic enzymes^42^, our evidence is that JMJD5 is a hydroxylase rather than a hydrolase; we observed no clear evidence for JMJD5-catalysed hydrolysis of the peptides we tested (within limits of detection). Thus, it is possible that the reported histone aminopeptidase activity of JMJD5^26,27^ requires factors other than the catalytic domain.

Although initially reported as a KDM^11^, JMJD5 lacks the extended flexible loop region, immediately N-terminal to βI, which folds into the active site and which is involved in binding *N*^ε^-methylated lysine residues in most, but not all, JmjC KDMs^1^ (Fig. 5). In addition, JMJD5 catalysis involves substantial active-site rearrangements and conformational changes in loop regions (Fig. 4); such movements have not been observed around the JmjC KDM active sites on *N*^ε^-methylated lysine binding but do occur, at least with some, JmjC-hydroxylases^30^ (Fig. 5). These structural features, together with the striking similarity in the geometric positioning of hydroxylated C-3 hydrogens between JMJD5 and FIH (Fig. 6e, f), and the lack of characteristic chromatin- and Zn-binding domains (present in most JmjC KDMs, Fig. 5), support our assigned hydroxylase, rather than KDM activity for JMJD5^1^.

The biological significance of JMJD5-catalysed arginyl-hydroxylation remains to be established. We have not yet been able to demonstrate cellular arginyl-hydroxylation of RPS6 or RCCD1 by endogenous JMJD5, in the former case because of the difficulties in observing sufficiently high quality proteomic coverage in the relevant region due to the abundance of charged residues in the vicinity of the target R137. In the case of RCCD1, we did not observe Arg-hydroxylation on the immuno-purified Myc-tagged RCCD1 from 293 T cells by proteomic analyses, possibly because RCCD1 is a relatively poor substrate for JMJD5 (Supplementary Fig. 8), or because hydroxylated RCCD1 undergoes rapid degradation. Our cellular results with JMJD5 contrast with those for ycfD, where we were able to obtain high quality MS results both for the intact ribosomal protein L16 and after proteolytic digestion^31^, demonstrating C-3 arginyl-hydroxylation does not preclude MS analyses. However, L16 is a relatively tractable protein compared to RCCD1 and RPS6; RCCD1 associates with chromatin (and with JMJD5)^20^ and RPS6 is subject to multiple PTMs (reviewed in refs.^43,44^). Importantly, it should be noted that even when using synthetic peptides, our MS/MS data for arginyl C-3 hydroxylation was not unequivocal (Fig. 1b) and required analyses by NMR and, following hydrolysis, by amino acid analysis and comparison with synthetic standards, for confident assignment of arginyl C-3 hydroxylation. Thus, the results imply a gap in the capability of current proteomics methodology and reaffirm the importance of comparison with synthetic standards for the assignment of new PTMs.

The evidence for human Arg-hydroxylation is notable as it defines a new type of mammalian enzyme-catalysed PTM that links the JmjC-hydroxylases and KDMs. Future work can focus on the role of JMJD5-catalysed Arg-hydroxylation in the regulation of translation and ribosome stability/function, where RPS6 has essential roles. JMJD5-catalysed RCCD1 Arg-hydroxylation may have a role in chromosome segregation as both JMJD5 and RCCD1 are required for the maintenance of chromosomal stability/fidelity during cell division^20^. Consistent with its role in cell proliferation, JMJD5 is substantially upregulated in cancers and leukaemia and consequently is a potential cancer target^22,23,45^. The unusual 2OG and substrate binding modes adopted by JMJD5 suggest that it should be possible both to identify selective inhibitors for JMJD5 and to avoid JMJD5 when targeting other human 2OG-oxygenases; the latter is important because inhibitors for human 2OG-oxygenases are in clinical trials for diseases including anaemia^46^ and cancer^9^.

## Methods

### Materials

Chemicals were from Sigma-Aldrich, Merck Chemicals or Alfa Aesar. Matrix-assisted laser desorption/ionisation time-of-flight (MALDI-TOF) matrices, matrix buffers and calibrants were from LaserBio Labs (Valbonne, France). RCCD1 (^139^CARAY^143^) and the RPS6 (^129^VPRRLGPKRASRIRKL^144^) peptide substrates (C-terminal amides) were either synthesised in-house (see below) or from G.L. Biochem (Shanghai, China). DNA primers were from Sigma Genosys.

### Expression and purification

DNA encoding for full-length JMJD5 (GI:223942014) was inserted into the pNIC28-Bsa4 vector (GenBank ID: EF198106) for expression in *E. coli* (ATCC 25922) with an N-terminal His_6_ tag. N-terminally truncated JMJD5 (aa 183–416) and (aa 153–416) constructs were generated by PCR from the full-length construct and inserted into pNIC28-Bsa4 and pNH-TrxT, respectively. JMJD5 variants were prepared using site-directed mutagenesis (New England Biolabs). Primer sequences are shown in Supplementary Table 2. All constructs were verified by DNA sequencing. Wild type and variant JMJD5 proteins were produced in *E*. *coli* Rosetta2 (DE3)-pLysS cells by induction with 0.5 mM isopropyl β-d-1-thiogalactopyranoside for 16–18 h at 18 °C (180 rpm). Cells were freeze-thawed and resuspended in 50 mM HEPES-Na pH 7.5, 500 mM NaCl, 20 mM imidazole (supplemented with an EDTA-free protease inhibitor cocktail tablet/Roche and bovine pancreatic grade II DNaseI/Roche) and lysed by ultra-sonication. Proteins were purified by Ni^2+^-affinity (5 mL HisTrap, GE Healthcare) chromatography followed by size-exclusion chromatography (50 mM HEPES-Na pH 7.5, 100–200 mM NaCl and 5% glycerol) and/or buffer-exchanged into storage buffer (50 mM HEPES-Na pH 7.5, 100–200 mM NaCl and 5% glycerol) using a 30 kDa MWCO filter (Amicon). KDM4A/JMJD2A (aa 1–359/ pNIC28-Bsa4) and ycfD (aa 1–373/pET-28a) were produced as described^29,30^. Protein purity was assessed by SDS-PAGE and masses of the purified proteins were verified by LC-MS using a Merck Chromolith C18 2 × 5 mm guard column coupled to a Waters LCT Premier XE, equipped with an electrospray interface.

### Peptide synthesis

All peptides in this study (except as described in the Materials section), including those in the ribosomal peptide library (Supplementary Data 1), were prepared by standard solid-phase synthesis using an Intavis Multipep automated peptide synthesiser with Tentagel-S-RAM resin (Rapp-Polymere) and *N*,*N*′-diisopropylcarbodiimide (DIC) as a coupling reagent. Final cleavage using 2.5% triisopropylsilane in CF_3_COOH yielded the peptides as C-terminal amides, which were precipitated in cold ether, redissolved in MilliQ water and then freeze-dried. The masses of peptide products were verified using MALDI-TOF mass spectrometry as described below.

### Endpoint assays

The hydroxylase/demethylase activityies of JMJD5 and JMJD2A on peptides was assessed using a MALDI-MS-based assay. Reactions consisted of enzyme (1–10 μM), (NH_4_)_2_Fe(SO_4_)_2_ (100 μM), sodium-(+)-l-ascorbate (500 μM), 2OG-disodium (200 μM) and peptide (10–50 μM) in 50 mM HEPES pH 7.5, 50 mM NaCl. Solutions of cofactors (ascorbate, 2OG and FeII) were prepared immediately prior to use at 20× the desired final concentration; ascorbate and 2OG were dissolved in 50 mM HEPES pH 7.5 and (NH_4_)_2_Fe(SO_4_)_2_ was prepared as a 100 mM solution in 20 mM HCl and diluted further with MilliQ water. Reactions were prepared by the addition of buffer and 2OG, followed by ascorbate, iron, enzyme and finally peptide, and were incubated at room temperature, or at 37 °C, for the indicated times before being quenched with 0.1% formate.

To assess product formation, 1 µL of quenched hydroxylation/demethylation reaction was mixed with 1 μL of α-cyano-4-hydroxycinnamic acid (CHCA) matrix (10 mg mL^−1^ in 50% acetonitrile, 50% MilliQ water with 0.1% trifluoroacetic acid) and spotted onto a MALDI plate for analysis using a MALDI micro-MX mass spectrometer (Waters, USA) in reflectron-positive ion mode: pulse voltage 1250 V, detector voltage 2750 V, mass suppression 1000 Da. Data were analysed using MassLynx 4.0.

### Kinetic analyses

For kinetic measurements, hydroxylation activity was tested using a mixture of (final concentrations) 10 µM JMJD5, 100 µM Fe(NH_4_)_2_(SO_4_)_2_ and 400 µM sodium-(+)-l-ascorbate in 50 mM HEPES-Na pH 7.5. Reaction was initiated by adding a mixture of 100 µM RPS6_129–144_ peptide (VPRRLGPK**R**ASRIRKL) and 500 µM 2OG-disodium salt solution. Reactions were carried out in V-bottom 96-well plates (Microlate, Grenier, Germany) on a mixer (Thermomixer C, Eppendorf) at 37 °C and quenched by addition of 1% formate. The extent of hydroxylation was examined by MALDI-TOF using CHCA as the matrix^34^. For the determination of the apparent *K*_m_ values, MALDI-MS-based assays were carried out over a range of Fe (3–150 µM)/2OG (3–150 µM)/RPS6_129–144_ (3–300 µM) concentrations. The initial reaction rates (ν_0_) for each concentration point were plotted against the corresponding Fe/2OG/RPS6 concentration using GraphPad Prism version 5.0 (GraphPad Software, San Diego, CA, USA) and the kinetic parameters (*K*_m_ and k_cat_) were determined based on the Michaelis–Menten equation.

### NMR analyses

To generate hydroxylated-RPS6_129–144_ peptide for NMR analysis, a large-scale (5 mL) reaction was prepared with 210 µM RPS6_129–144_ peptide (~2 mg total), 40 µM His_6_-JMJD5, 1 mM ascorbate, 400 µM (NH_4_)_2_Fe(SO_4_)_2_ and 400 µM 2OG. The reaction was incubated at room temperature and product formation monitored at 10-min intervals by MALDI-TOF MS. After 1 h, 100% of the peptide had been converted to the hydroxylated product. Hydroxy-RPS6 was purified by gel filtration using a Superdex Peptide 10/300 GL column (GE Healthcare) in 200 mM ammonium bicarbonate, then desalted using a Sep-Pak C18 cartridge (Waters, USA). The purified peptide was freeze dried and stored at −20 °C.

Cofactors were prepared as described above in 20 mM Tris pH 7.5. Recombinant His_6_-JMJD5 proteins (wild type and H321A variant) were buffer exchanged into 20 mM Tris pH 7.5 using Micro Bio-Spin Chromatography Columns (Bio-Rad). One hundred sixty-microlitre reactions were prepared in a 1.5 mL Eppendorf tube with 16 µL D_2_O, 100 µM (NH_4_)_2_Fe(SO_4_)_2_, 200 µM 2OG, ±500 µM ascorbate, ±1 mM NOG and ±10 µM His_6_-JMJD5 (wild type/H321A) in 20 mM Tris D_11_ (Cambridge Isotope Laboratories), pH 7.5. Reactions were initiated by the addition of enzyme and immediately transferred to a 3 mm Shigemi NMR tube, which was centrifuged for a few seconds in a hand centrifuge. Proton NMR spectra were acquired, and the reaction was monitored in real time using a Bruker Avance III 700 MHz spectrometer equipped with a 5 mm inverse TCI cryoprobe at 298 K. The AV700 was controlled by TopSpin software; spectrometer conditions were optimised using a control sample with all reaction components except enzyme prior to data acquisition. The first proton spectrum was acquired with water suppression, 325 s after mixing, following brief optimisation (start of data acquisition was 225 s). Spectra were automatically acquired every 105 s for 1900 s. TopSpin software was used to process and integrate the peaks corresponding to 2OG and succinate for analysis using Microsoft Excel.

Structure determination of the hydroxylated product was carried out on a sample containing the hydroxylated peptide in D_2_O by NMR, using a Bruker Avance AVIII 700 MHz NMR spectrometer equipped with an inverse TCI cryoprobe, optimised for ^1^H observation, and installed with Topspin software. The D_2_O deuterium signal was used as an internal lock signal and ^1^H chemical shifts are reported relative to the solvent (δ_H_ 4.7 ppm). Phase-sensitive HSQC and magnitude-mode COSY spectra used pulsed field gradients and phase-sensitive TOCSY experiments used the MLEV-17 mixing scheme (mixing times = 120 ms).

### Amino-acid analyses

For the amino-acid analysis, 6-aminoquinolyl-*N*-hydroxysuccinimidyl carbamate (AQC) was synthesised following the procedure of Cohen and Michaud^47^. Di-(*N*-succinimidyl) carbonate (DSC) (0.213 g; 0.832 mmol; 1.2 eq.) was dissolved in dry acetonitrile (7 mL) under a N_2_ atm, and heated to reflux. 6-Aminoquinoline (0.107 g; 0.694 mmol; 1 eq.) dissolved in dry acetonitrile (3 mL) was added dropwise to the refluxing solution over 30 min. The solution was refluxed for another 30 min, during which formation of a yellow precipitate was observed. The solution was then allowed to cool to room temperature and the yellow precipitate was filtered off before concentrating to half volume, and cooling to 4 °C overnight to allow the product to precipitate. The resulting white crystals were collected by filtration, and washed with cold acetonitrile. The product was recrystallised from acetonitrile (52% yield) and characterised by comparison of its NMR data and melting point with those reported^47^.

Hydroxylated RPS6_129–144_ peptide for amino-acid analysis was prepared as described^48^. Dried reaction mixtures (containing ~20 µg of hydroxylated peptide) were reconstituted in MilliQ water and enzymatic hydrolysis was carried out for 16 h at 37 °C using protease from *Streptomyces griseus* at a 1:5 protease-to-peptide ratio. Hydrolysates were dried by vacuum centrifugation, reconstituted in 80 µL of 0.2 M borate buffer pH 9.0 and derivatised by the addition of 20 µL AQC. Hydroxyarginine standards used to assign stereochemistry (2*S*,3*R*-Arg(OH)•2HCl and 2*S*,3*S*-Arg(OH)•2HCl) were kind gifts from Christian Ducho, Georg-August-University Göttingen^49^.

### Mass spectrometric analyses

LC-MS analyses of derivatised amino-acid standards and hydrolysates were performed as described^50^ using a Waters Acquity ultra performance liquid chromatography system coupled to an LCT Premier XE orthogonal acceleration time-of-flight mass spectrometer equipped with an electrospray ionisation source (Waters, USA). Derivatised amino acids were separated using an increasing gradient of Solvent B (1.3% (v/v) formic acid in acetonitrile) in Solvent A (AccQ Tag Ultra Phase A), at a flow rate of 0.7 mL min^−1^. The conditions for ESI-MS detection were as follows: positive-ion mode, V-mode analyser, desolvation temperature at 250 °C, source temperature at 120 °C, capillary voltage at 3000 V, sample cone voltage at 100 V, cone and desolvation gas flow at 30 and 550 L min^−1^, respectively. The MS data were acquired and an extracted ion chromatogram produced for *m*/*z* 361, corresponding to singly derivatised hydroxyarginine. Data were analysed using MassLynx 4.1.

For tandem MS analyses, samples were directly mixed onto the MALDI target plate using 0.8 µL of 10 mg mL^−1^ CHCA in 50% (v/v) acetonitrile with 0.1% (v/v) formic acid and air-dried. Tandem MS was performed using a MALDI-TOF/TOF 4800 plus mass spectrometer (Applied Biosystems). Each reflectron MS spectrum was collected in an independent acquisition positive mode, typically using 1000 laser shots per spectra and a fixed laser intensity of 2900 V. The strongest precursors were selected for MS/MS, and the analyses were performed using Collision Induced Dissociation (CID) assisted with air, with a collision energy of 1 kV and gas pressure of 1 × 106 torr. Two thousand laser shots were collected for each MS/MS spectrum using a fixed laser intensity of 4500 V. Raw data analysis was performed with Data Explorer 2.1 software from Applied Biosystems. The mass corresponding to *x* and *x* + 16, equivalent to one hydroxylation, were identified and MS/MS spectra were annotated manually.

### Crystallography

Crystals of N-terminally truncated JMJD5 (aa 153–416 and aa 183–416) wt/variant complexes (0.8 mM His_6_-JMJD5, 1.5 mM MnCl_2_, 2.5 mM 2OG/NOG (or 50 mM succinate) and 10 mM substrate) were grown by vapour diffusion at 22 °C in 300 nL sitting drops in 2:1 or 1:1 or 1:2 ratio of sample to well solution (0.1 M Bis-Tris pH 5.8–6.5, 15–32% w/v polyethylene glycol 3350, 2 mM MnCl_2_). In general, crystals were cryoprotected by transferring to a solution of mother liquor supplemented with 25% (v/v) glycerol before being cyro-cooled in liquid N_2_. Data were collected at 100 K using synchrotron radiation at the Diamond Light Source (DLS) beamline I03 (0.9795 Å) equipped with a Dectris Pilatus3 6M detector. Data were processed as outlined in the Supplementary Tables 4 and 5. Structures were solved by molecular replacement using PHASER^51^ (search model PDB ID 4GJZ)^24^ and refined by alternative cycles of PHENIX^52^, CNS^53^ and BUSTER^54^ using the maximum-likelihood function and bulk-solvent modelling. Iterative cycles of model building in COOT^55^ and refinement proceeded until the *R*/*R*_free_ values converged. Final rounds of refinement were performed by PHENIX^52^. MOLPROBITY^56^ was used to monitor the geometric quality of the models between refinement cycles and identify poorly modelled areas needing attention. Water molecules were added to peaks >1.5σ in 2*F*_o_−*F*_c_ electron density maps that were within hydrogen bonding distance to protein residues with reasonable hydrogen bonding geometry. Data collection and refinement statistics are shown in the Supplementary Tables 4 and 5.

### Statistical analysis

Endpoint assay results are the mean of three independent experiments with error bars representing the s.e.m. For kinetic measurements, each experiment was carried out (at least) in triplicate (*n* = 3–9).

### Data availability

GenBank accession codes for the sequences mentioned in this article are as follows: Q8N371 (KDM8_HUMAN); P62753 (RS6_HUMAN); A6NED2 (RCCD1_HUMAN). Atomic coordinates and structure factors for the crystal structures of JMJD5-apo (PDB: 6F4M), JMJD5.2OG (PDB: 6F4N), JMJD5.succinate (PDB: 6F4O), Complexes 1 (PDB: 6F4P), 2 (PDB: 6F4Q), 3 (PDB: 6F4R), 4 (PDB: 6F4S), and 5 (PDB: 6F4T) are deposited in the protein databank (wwPDB). Additional data supporting the findings of this study are available from the corresponding authors on reasonable request.

## Electronic supplementary material

Supplementary Information(PDF 2512 kb)

Description of Additional Supplementary Files(PDF 170 kb)

Supplementary Data 1(XLSX 802 kb)
