# Publisher Correction: JMJD5 is a human arginyl C-3 hydroxylase

**DOI:** 10.1038/s41467-018-04196-7

**Published:** 2018-04-23

**Authors:** Sarah E. Wilkins, Md. Saiful Islam, Joan M. Gannon, Suzana Markolovic, Richard J. Hopkinson, Wei Ge, Christopher J. Schofield, Rasheduzzaman Chowdhury

**Affiliations:** 1 The Department of Chemistry, University of Oxford, Mansfield Road, Oxford, OX1 3TA UK; 2 Present Address: Leicester Institute of Structural and Chemical Biology and Department of Chemistry, University of Leicester, Lancaster Road, Leicester, LE1 7RH UK; 3 Present Address: Stanford University School of Medicine, Department of Molecular and Cellular Physiology, Clark Center, Stanford, CA 94305-5345 USA

Correction to: *Nature Communications* 10.1038/s41467-018-03410-w, published online 21 March 2018

The originally published version of this Article contained an error in the spelling of the author Md. Saiful Islam, which was incorrectly given as Saiful Islam. This has now been corrected in both the PDF and HTML versions of the Article.

