# Supplementary material for: JMJD5 is a human arginyl C-3 hydroxylase
Source: Nat Commun. 2018 Mar 21;9:1180. doi: 10.1038/s41467-018-03410-w (PMC5862942; doi:10.1038/s41467-018-03410-w)
Supplement: Supplementary file 1 — Supplementary Information(PDF 2512 kb) [file 41467_2018_3410_MOESM1_ESM.pdf]

SUPPLEMENTARY INFORMATION

JMJD5 is a Human Arginyl C-3 Hydroxylase

Wilkins et al.

Supplementary Figures

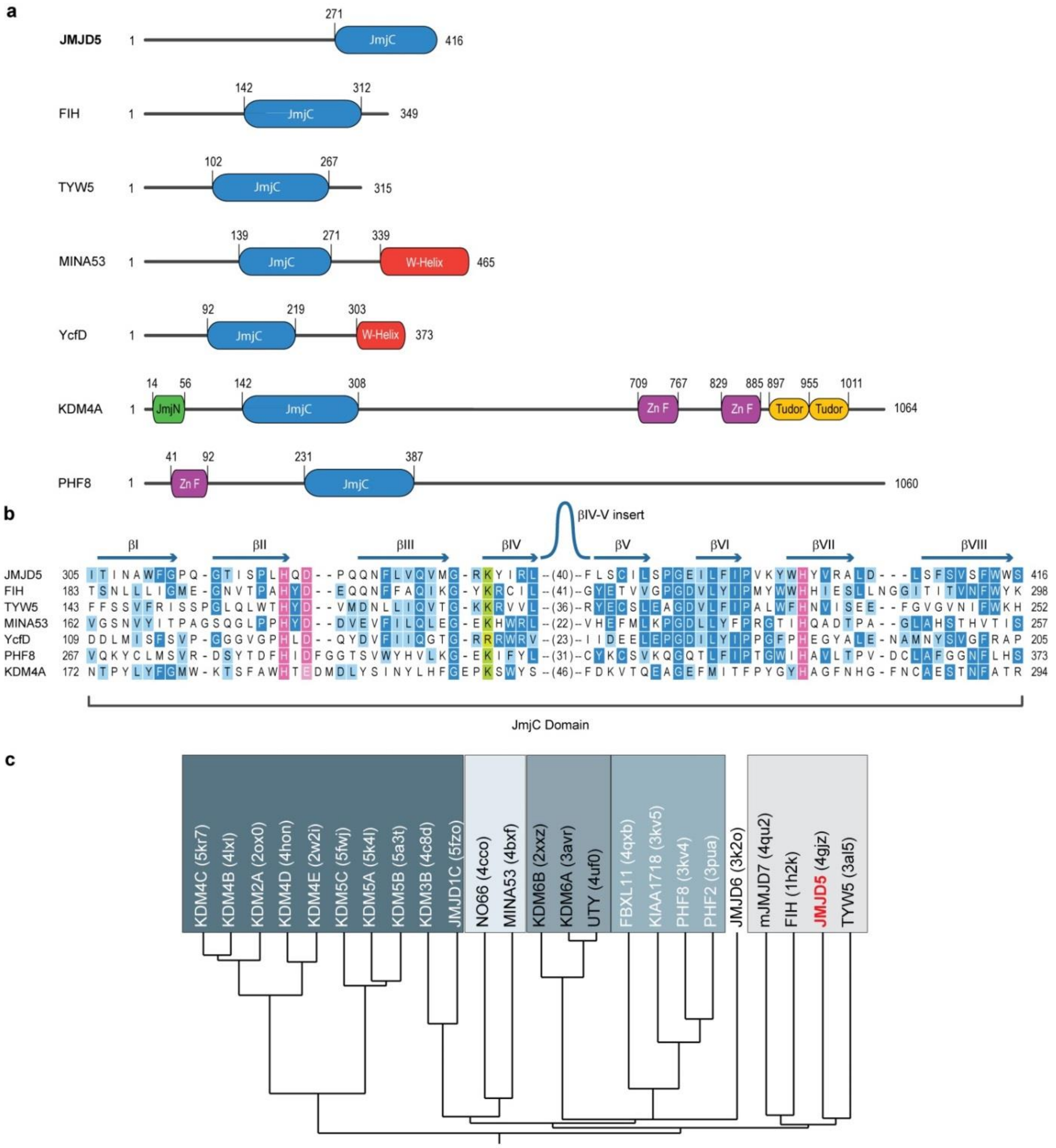

Supplementary Figure 1 | Domain architecture, sequence alignment and phylogenetic analysis of JMJD5 and related JmjC enzymes. **a**, Domain architecture of JMJD5 and related JmjC-oxygenases. **b**, Amino acid sequence alignment of the JmjC domains from JMJD5 (NP_079049), the tRNA hydroxylase TYW5 (NP_001034782), protein hydroxylases FIH (NP_060372), MINA53 (NP_001035998) and ycfD from *E. coli* (NP_415646), and the lysine demethylases PHF8 (KDM7B, NP_001171825) and KDM4A (NP_055478). Box-shading highlights Fe(II) (pink) or 2OG (green) binding residues and those that are conserved (dark blue) or chemically similar (light blue) in at least 3 proteins. Arrows above the alignment show the positions of DSBH β-strands in JMJD5 (βI-VIII). **c**, Phylogenetic analyses of JMJD5 and related JmjC-oxygenase structures (PDB codes in the parentheses) imply JMJD5 is more similar to JmjC-hydroxylases than the KDMs. The most similar JmjC-hydroxylases to JMJD5 are (in the order of decreasing similarity): the tRNA hydroxylase TYW5 (PDB: 3AL5[1], Cα RMSD=1.9, Z=26.2), the histone aminopeptidase JMJD7 (PDB: 4QU2, Cα RMSD=1.9, Z=25.3), the asparaginyl hydroxylase FIH (PDB: 1H2K[2], Cα RMSD=2.2, Z=24.4), and the lysyl hydroxylase JMJD6 (PDB: 3K2O[3], Cα RMSD=2.8, Z=21.3).

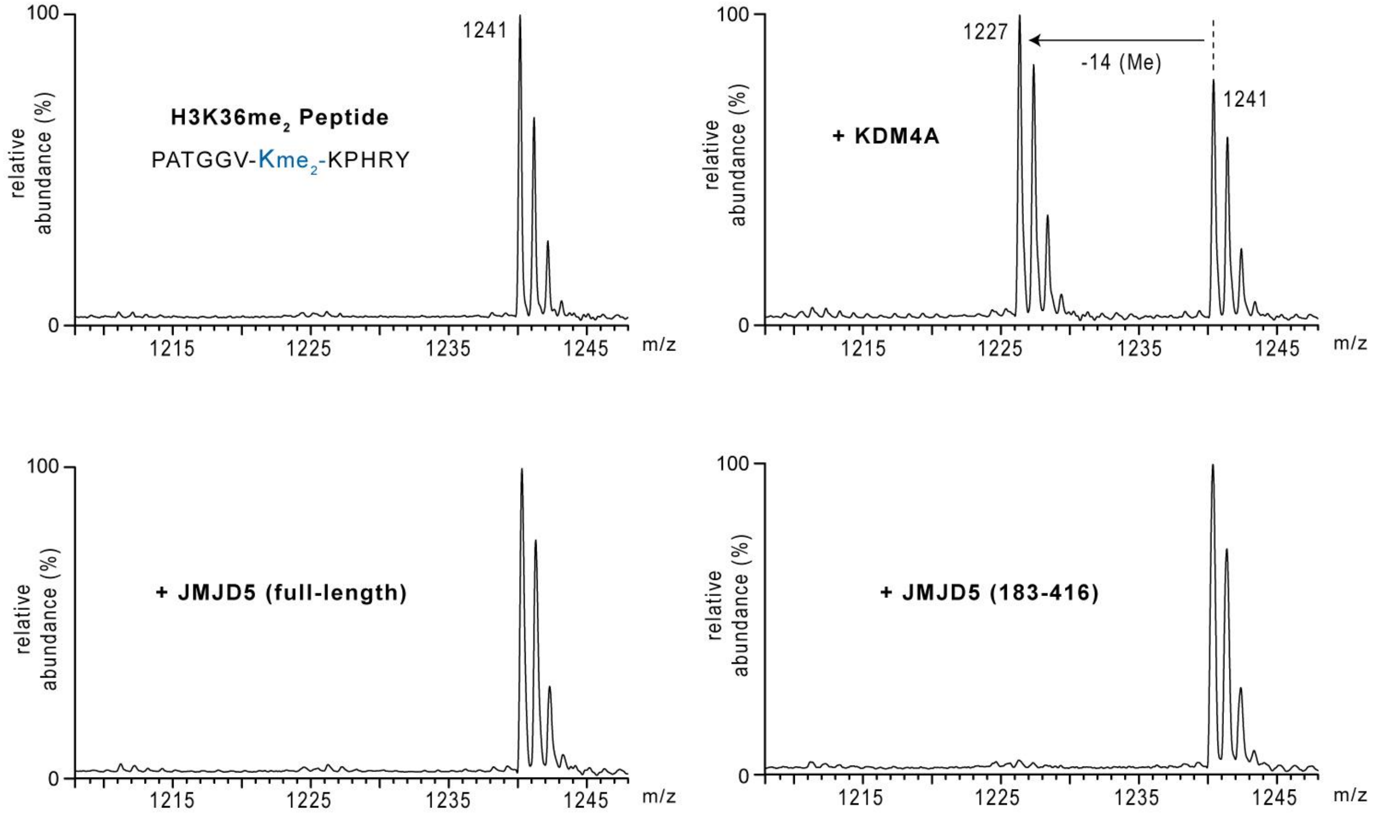

Supplementary Figure 2 | Evidence that JMJD5 is not a KDM. Representative MALDI-MS spectra of a synthetic histone H3K36me$_2$ peptide treated with Fe(II), 2OG, ascorbate and either JMJD5$_{1\text{-}416}$, JMJD5$_{183\text{-}416}$ or KDM4A$_{1\text{-}359}$ as indicated. A peak shift of -14 (corresponding to loss of a methyl group) is observed following treatment with KDM4A but not JMJD5.

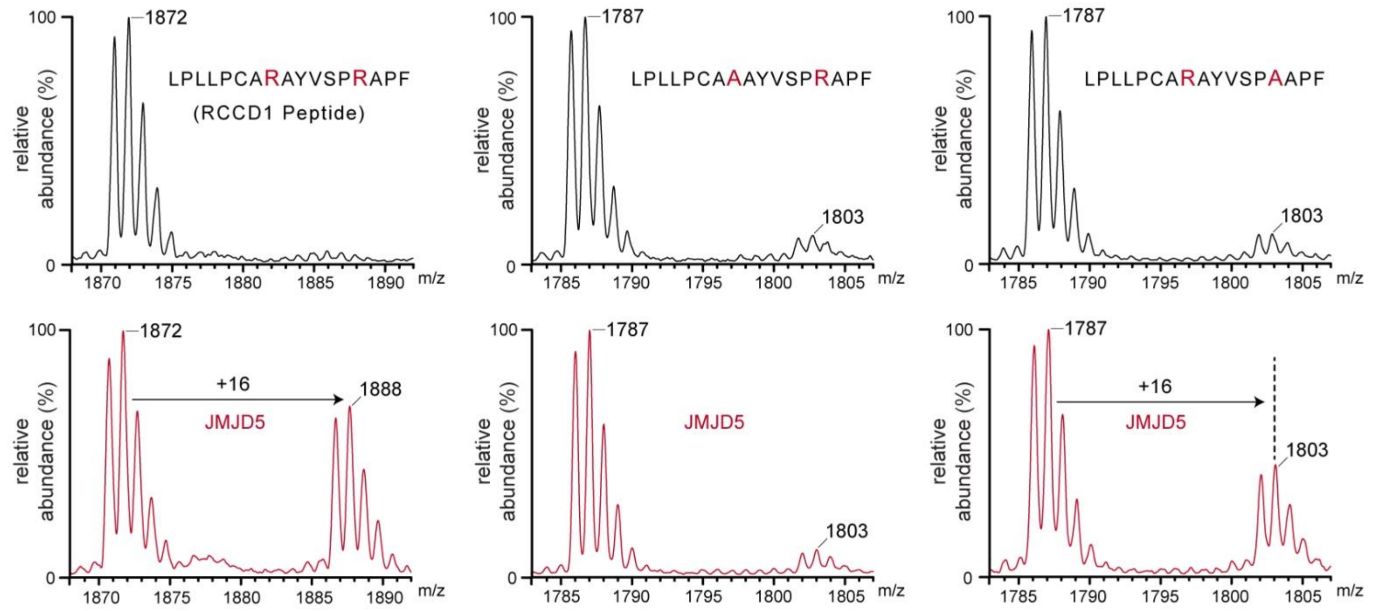

Supplementary Figure 3 | RCCD1 peptides are substrates for JMJD5-catalysed arginyl-hydroxylation. Representative MALDI-MS spectra of synthetic peptides from RCCD1 treated with JMJD5, 2OG and Fe(II) (red) or cofactors alone (black). Peptides correspond to the wildtype RCCD1 sequence (residues 134-150, RefSeq ID: NP_001017919) or variants thereof, in which one of the two arginine residues is substituted with an alanine residue (R141A or R147A).

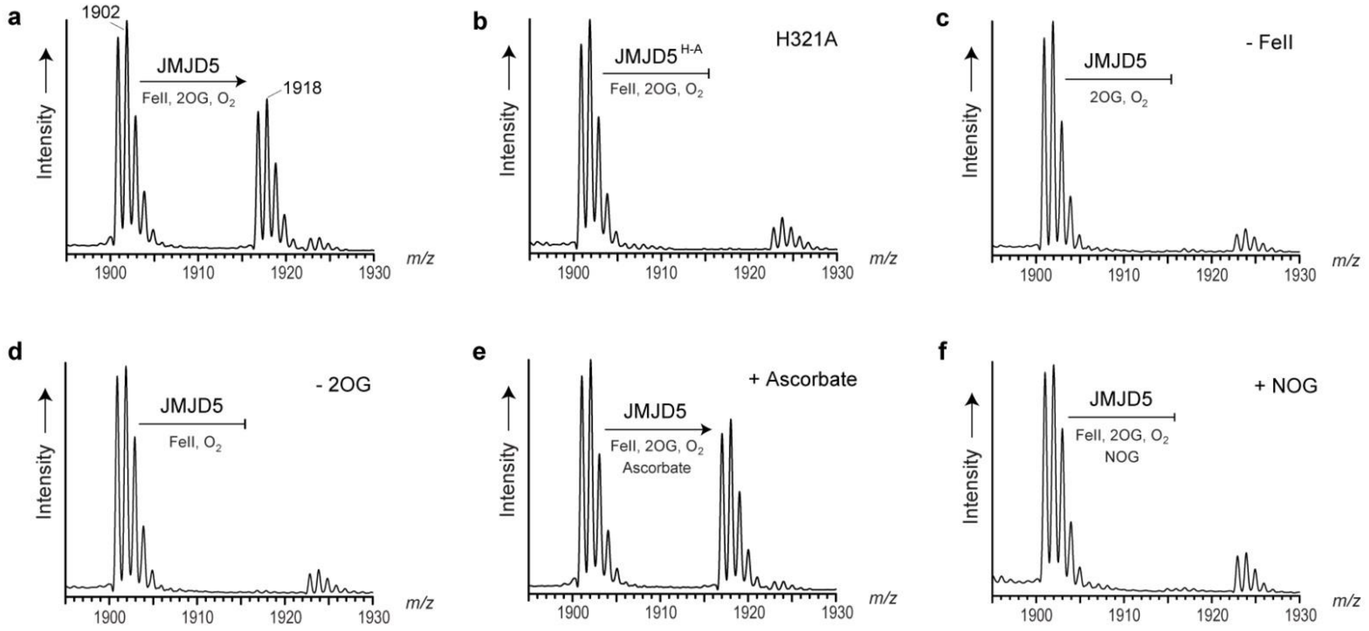

Supplementary Figure 4 | RPS6 peptides are substrates for JMJD5-catalysed arginyl-hydroxylation. Representative MALDI-MS spectra of hydroxylation assays with 5 μM His$_6$-JMJD5$_{1-416}$, 50 μM RPS6 peptide, 100 μM Fe(II) and 200 μM 2OG**.** **a**, contains all reaction components, **b**, contains the H321A variant in place of wildtype JMJD5, **c**-**f** include all components except for Fe(II) (**c**) and 2OG (**d**) and/or with the addition of 1 mM ascorbate (**e**) or the 2OG competitor, NOG (**f**). Reactions were at 22 °C and quenched after 10 minutes by addition of 1% formate. Addition of ascorbate had little effect on JMJD5-catalysed hydroxylation of the RPS6 peptide, although it promoted a small increase in 2OG turnover by JMJD5 after longer incubation times.

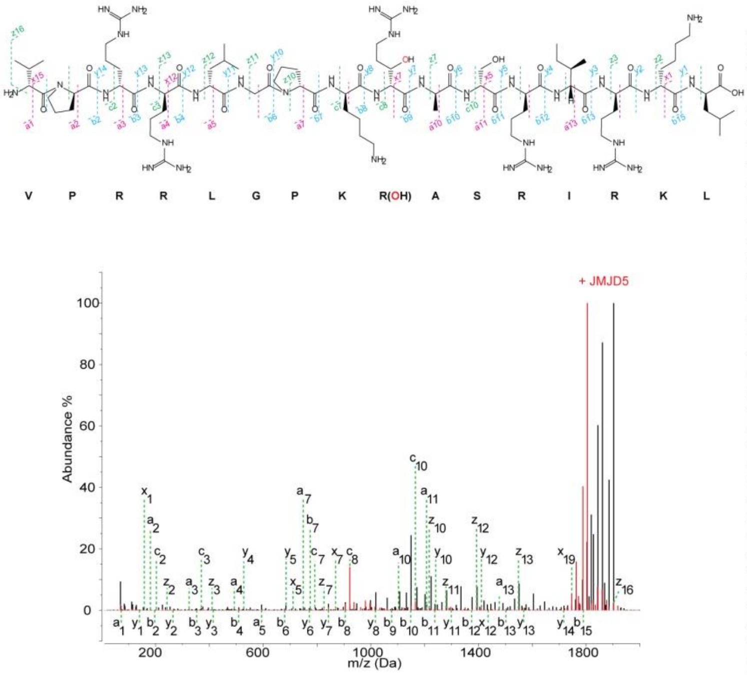

1902.45	Predicted m/z	Observed m/z	Deviation
a1	72.081	72.102	-0.021
z1	114.097	114.128	-0.031
y1	131.118	131.129	-0.011
x1	157.101	157.110	-0.009
a2	169.134	169.138	-0.005
b2	197.129	197.143	-0.015
c2	214.155	214.210	-0.055
z2	242.191	242.180	0.012
x2	285.196	285.187	0.009
a3	325.235	325.228	0.006
b3	353.230	353.222	0.007
c3	370.256	370.244	0.013
y3	415.314	415.310	0.004
a4	481.336	481.312	0.024
b4	509.331	509.303	0.028
y4	528.398	528.406	-0.008
a5	594.420	594.472	-0.053
z5	667.478	667.441	0.036
b6	679.436	679.677	-0.241
y5	684.499	684.491	0.008
a7	748.494	748.468	0.026
y6	771.531	771.285	0.246
c7	793.516	793.515	0.001
y7	842.568	842.498	0.070
a8	876.589	876.566	0.023
b8	904.584	904.524	0.060
y8	998.669	998.657	0.012
b9	1060.685	1060.629	0.056
b10	1131.722	1131.719	0.003
c10	1148.749	1148.756	-0.007
x9	1152.748	1152.189	0.558
a11	1190.759	1190.586	0.174
z10	1206.796	1206.809	-0.013
b11	1218.754	1218.729	0.025
y10	1223.817	1223.808	0.009
z11	1263.817	1263.889	-0.072
y11	1280.839	1280.854	-0.015
b12	1374.855	1374.817	0.038
z12	1376.901	1376.941	-0.040
y12	1393.923	1393.901	0.022
x12	1419.906	1419.981	-0.075
a13	1459.944	1460.061	-0.116
c13	1504.966	1505.377	-0.411
z13	1533.002	1533.066	-0.064
a15	1744.141	1744.355	-0.214
c15	1789.163	1789.234	-0.071
y15	1803.178	1803.734	-0.556
z16	1885.225	1884.697	0.528

1918.45 Da	Predicted m/z	Observed m/z	Deviation
a1	72.081	72.100	-0.019
y1	131.120	131.104	0.016
x1	157.101	157.115	-0.014
a2	169.134	169.132	0.002
b2	197.129	197.130	-0.002
c2	214.155	214.142	0.013
z2	242.191	242.286	-0.095
y2	259.213	259.181	0.032
a3	325.235	325.228	0.007
b3	353.230	353.213	0.017
c3	370.256	370.227	0.029
z3	398.293	398.314	-0.022
y3	415.314	415.282	0.032
a4	481.336	481.306	0.030
b4	509.331	509.311	0.019
y4	528.398	528.346	0.052
a5	594.420	594.393	0.027
b6	679.436	679.390	0.046
y5	684.499	684.460	0.039
x5	710.482	710.390	0.092
a7	748.494	748.499	-0.005
y6	771.531	771.437	0.094
b7	776.489	776.480	0.009
c7	793.516	793.539	-0.024
z7	825.547	825.541	0.006
y7	842.568	842.550	0.018
x7	868.552	868.473	0.079
b8	904.584	904.558	0.026
c8	921.611	921.570	0.041
y8	1014.659	1014.586	0.073
b9	1076.675	1076.591	0.084
a10	1119.717	1119.637	0.080
b10	1147.712	1147.674	0.038
c10	1164.739	1164.727	0.011
a11	1206.749	1206.971	-0.222
z10	1222.786	1222.714	0.072
b11	1234.744	1234.693	0.051
y10	1239.807	1239.740	0.067
z11	1279.807	1279.807	0.000
y11	1296.833	1296.746	0.086
b12	1390.845	1390.789	0.056
z12	1392.891	1392.908	-0.017
y12	1409.913	1409.860	0.053
x12	1435.896	1435.888	0.008
a13	1475.934	1475.776	0.158
b13	1503.929	1503.984	-0.055
z13	1548.992	1549.113	-0.120
y13	1566.014	1565.946	0.067
y14	1722.115	1722.557	-0.442
x15	1748.098	1748.462	-0.364
b15	1788.125	1788.363	-0.237
z16	1901.215	1900.763	0.452

Supplementary Figure 5 | MSMS spectra showing hydroxylation of an RPS6 peptide (16-mer) in the presence of Fe(II) and 2OG with (red) and without (black) JMJD5. MS/MS spectra showing JMJD5 catalysed hydroxylation of a $RPS6_{129\text{-}144}$ peptide in the presence of 2OG and Fe(II). The results imply but do not unequivocally demonstrate hydroxylation of the arginine corresponding to RPS6 R137, as supported by subsequent NMR and amino acid analyses (Fig. 1). The a, b, c, x, y, and z ions annotated in this spectrum were assigned manually using a fragment mass tolerance of 0.6 da. The m/z of fragments from a mass list generated from the spectrum were matched to predicted fragments from 'http://prospector.ucsf.edu/prospector/cgi-bin/msform.cgi?form=msproduct'. The noise filter smooth had a correlation factor of 0.7; noise removal had a correlation factor of 2 and a Gaussian smooth filter width of 5 points. The fragmented peptide sequence of resulting MS is shown as an insert. The tables show the predicted and observed masses of the identified fragments and deviations from predicted masses.

VPRRLGPKXASRIRKL

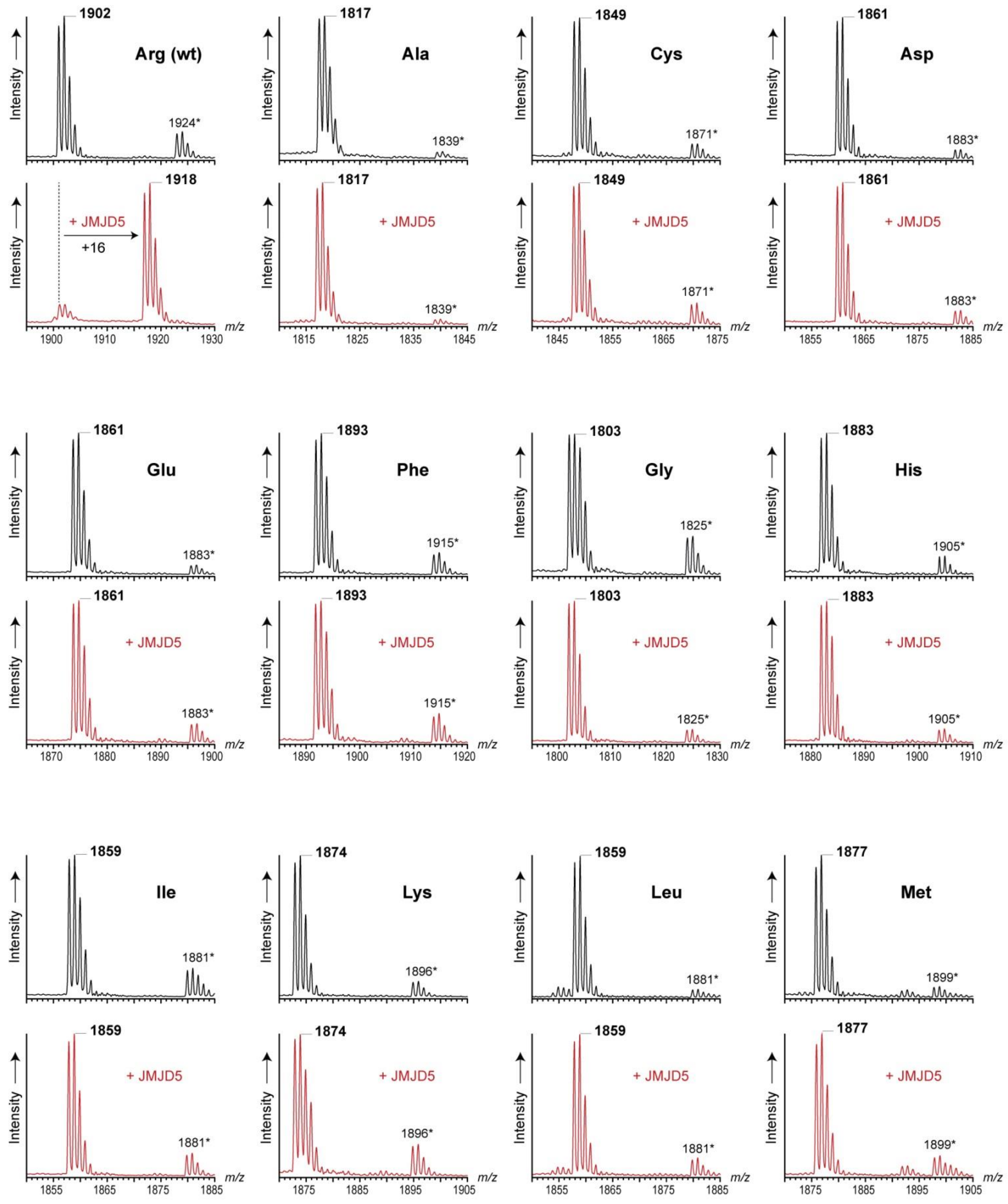

Contd.

V P R R L G P K X A S R I R K L

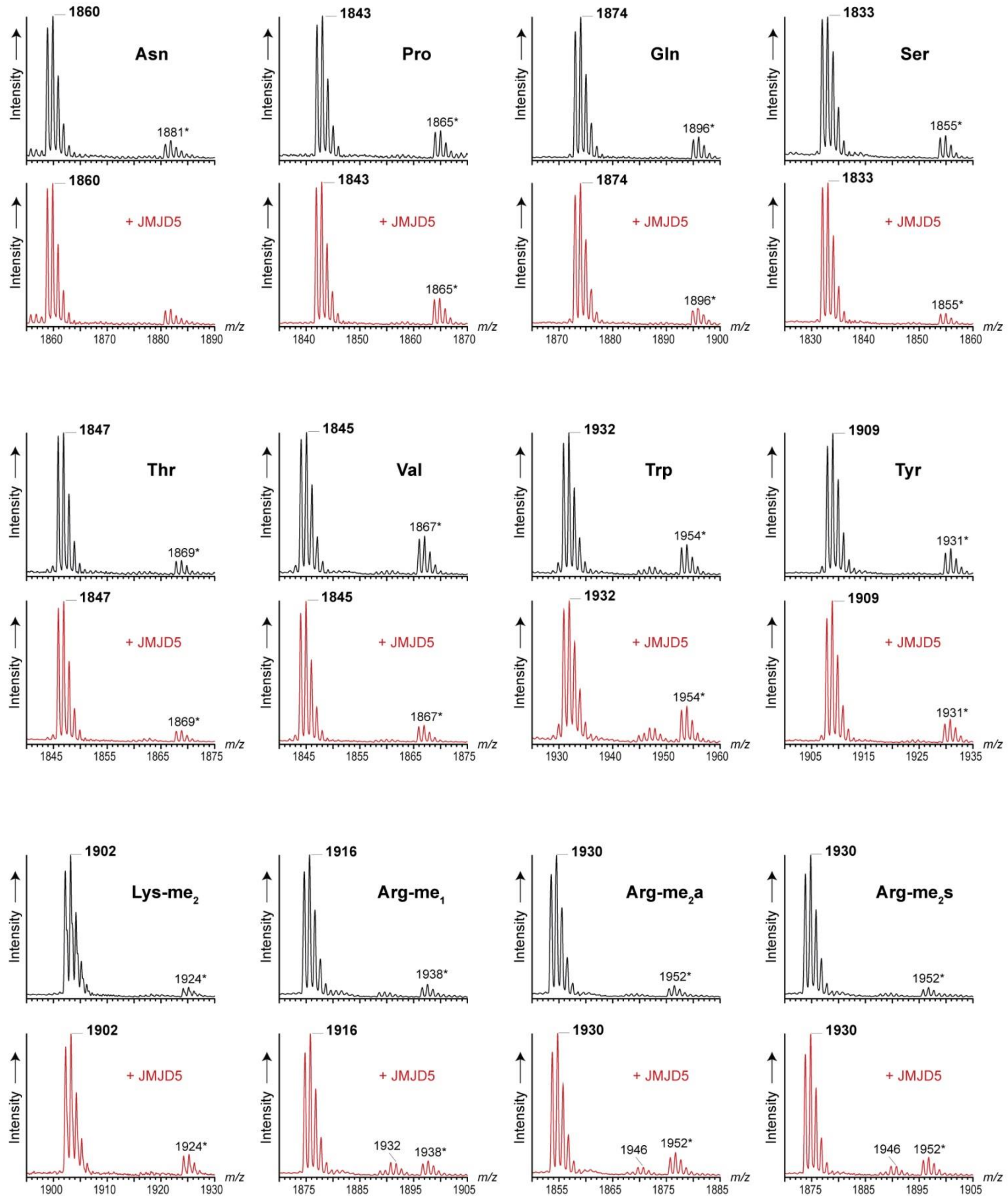

Supplementary Figure 6 | Evidence that JMJD5-catalysed hydroxylation is specific to arginine residues. Representative MALDI-MS spectra of synthetic peptides derived from $RPS6_{129-144}$ wherein the arginine residue hydroxylated by JMJD5 (R137) is substituted with other naturally occurring L-amino acids, dimethyl-lysine (Lys-me_2), mono-methyl arginine (Arg-me_1), symmetric or asymmetric di-methyl arginine (Arg-me_2s/me_2a). * denotes peaks corresponding to +22 (Na^+) adducts under MS conditions.

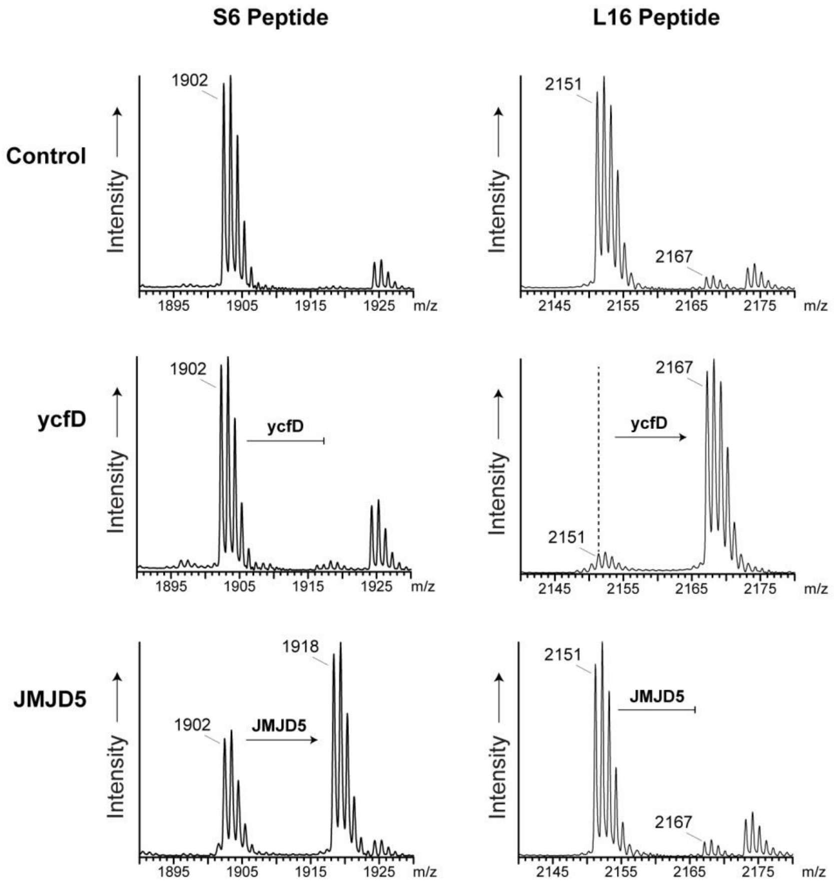

Supplementary Figure 7 | The arginyl-hydroxylases JMJD5 and ycfD do not exhibit any cross-reactivity. Because both JMJD5 and ycfD catalyse Arg-hydroxylation (on different substrates) with the same (3*R*)-stereochemistry, we tested whether there is any cross-reactivity. The figure shows representative MALDI-MS spectra for human $RPS6_{129\text{-}144}$ (left) or *E. coli* $L16_{71\text{-}90}$ (right) following treatment with JMJD5, ycfD or cofactors alone. A +16 *m/z* shift corresponding to a single hydroxylation is observed following treatment of the S6 peptide with JMJD5 but not ycfD. Likewise, the L16 peptide is hydroxylated by ycfD, but not JMJD5.

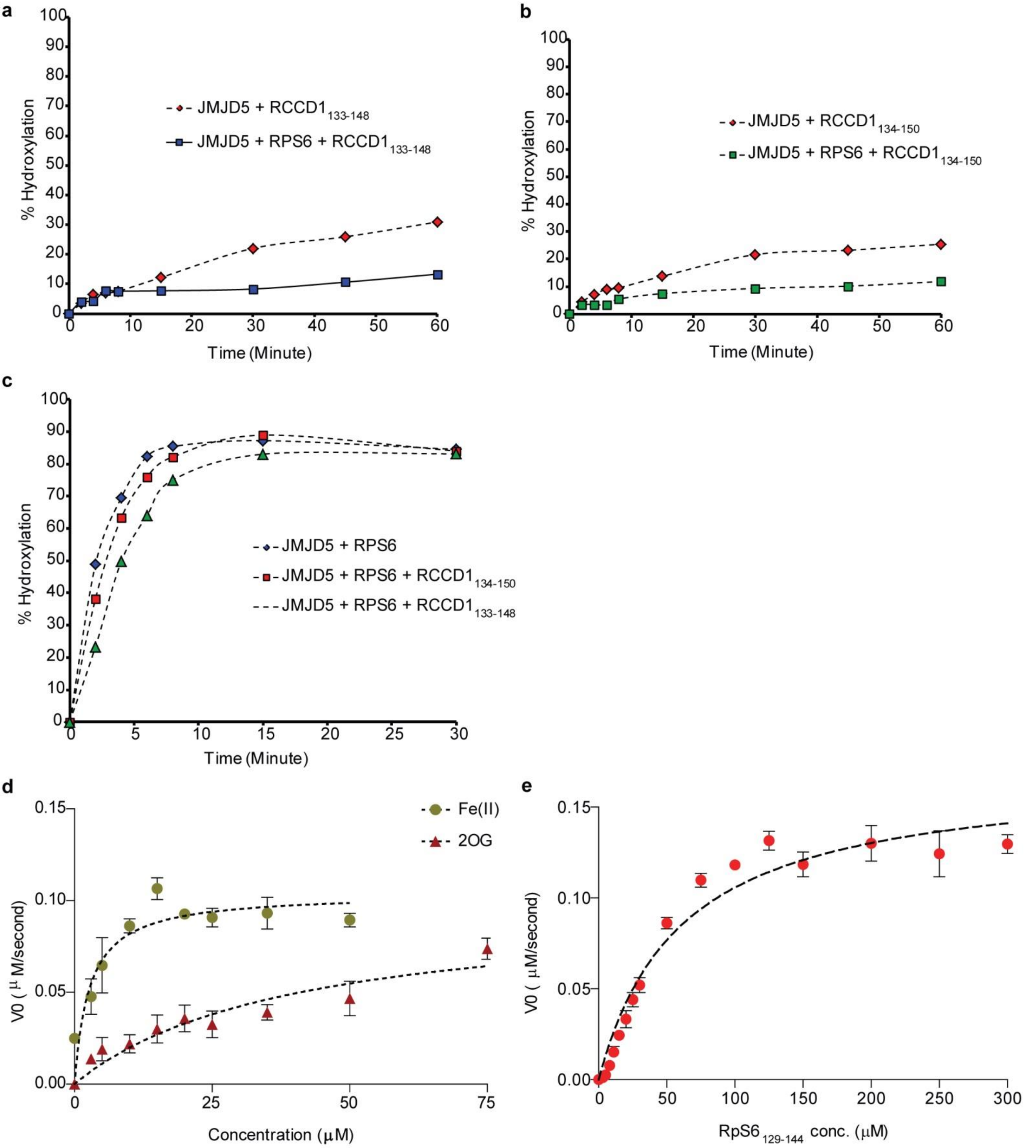

Supplementary Figure 8 | RPS6 is a preferred JMJD5 substrate. a-c, Results of the competition experiments where JMJD5 was incubated with equimolar amounts of $RCCD1_{134-150}$ (or $RCCD1_{133-148}$) and $RPS6_{129-144}$ show JMJD5 preferentially catalyses RPS6 peptide hydroxylation. MALDI-MS was used to analyse hydroxylation of RCCD1 (**a** and **b**) and RPS6 (**c**) using single or combination of peptides in the reaction mix as indicated. Figures **d** and **e** show kinetic analyses of Fe(II), 2OG and RPS6 for JMJD5-catalyzed RPS6 hydroxylation. The results reveal that JMJD5 has unusually low K_m for both Fe(II) (2.7 ± 0.7 µM, **d**) and 2OG (9.5 ± 2.0 µM, **d**) suggesting strong binding but relatively high K_m value for $RPS6_{129-144}$ peptide (60.4 µM) (compared to other JmjC enzymes). Values are mean ± s.e.m. (n = 3). See Methods for details.

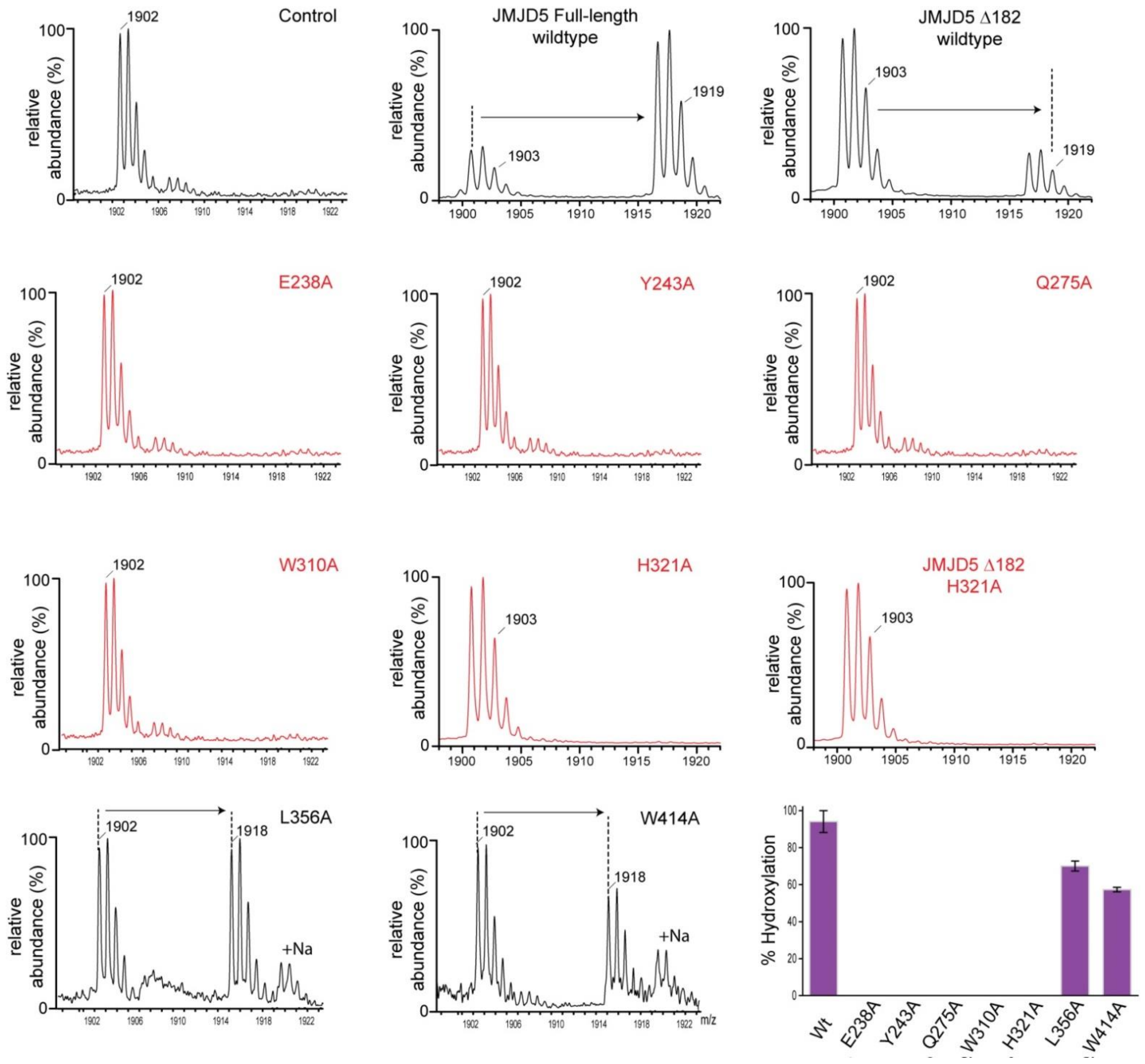

Supplementary Figure 9 | Variant analyses of the 2OG and substrate-binding residues located on the JmjC domain of JMJD5. Relative catalytic activities of wildtype and alanine-variants of residues around the active site pocket of JMJD5 and the corresponding representative MALDI-TOF MS spectra. All variants were produced using the JMJD5 full-length construct except JMJD5 Δ182. The JMJD5 *N*-terminus (aa 1-182) was reported to constitute an 'inhibitory domain' that was proposed to block its demethylase activity. The *N*-terminally truncated construct of JMJD5 (aa 183-416) was less active for RPS6 hydroxylation. In the JMJD5.substrate complexes, the hydroxylated arginine in both RPS6 and RCCD1 bind in a shallow channel on the JMJD5 surface forming multiple interactions/ hydrogen bonds with E238 and S318. The hydrophobic patches present along the exposed minor sheet including the β3-β4 loop (Y243), the βII-βIII loop (P324, Q325), and βVIII on the major sheet (W414) and the hairpin (L356, L357) are involved in substrate binding. In support of the importance of these interactions, substitution of E238, Y243, W310 and H321 by Ala led to complete loss of activity, while substitution of L356 and W414 with Ala led to 40-50% reduction of RPS6 hydroxylation. Results are the mean ± s.e.m. (n = 3). Assay conditions: 10 µM full-length human JMJD5, 100 µM $Fe(NH_4)_2(SO_4)_2$ and 400 µM (+)-sodium L-ascorbate were added in the reaction buffer 50 mM HEPES-Na pH 7.5. The reaction was initiated by adding the reaction mixture comprising 500 µM 2OG and 100 µM $RPS6_{129-144}$. See Methods for details.

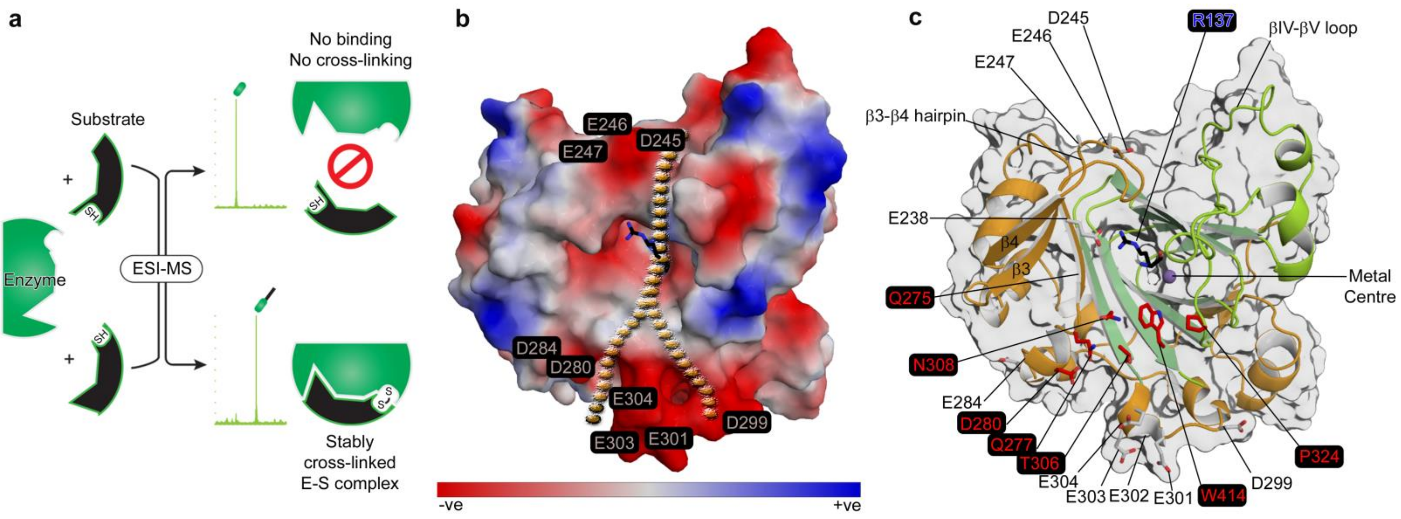

Supplementary Figure 10 | Chemical cross-linking strategy adopted to obtain stable JMJD5.substrate complexes for crystallisation. **a**, Outline of the 'Chemical cross-linking' strategy for stabilising labile protein-protein/-nucleic acid complexes[4, 5, 6, 7]. **b**, A potential substrate binding channel on the JMJD5 surface was identified using computational cleft analysis; the channel runs the length of the catalytic domain and is characterised by two acidic patches at either end formed by residues from the *N*-terminal extension of the DSBH. One end of the channel is formed by JMJD5 β3 (containing E238) and the β3-β4 loop containing 3 sequential acidic residues (D245, E246 and E247). The other end is embedded between two acidic patches composed of 3_{10} helix-2 (D280) and α4 (E284) on one side and 3_{10} helix-3 containing 5 acidic residues (D299, E301, E302, E303 and E304) on the other side. The long potential substrate binding channel with acidic ends is a distinctive structural feature of JMJD5 and is apparently well-suited to recognise positively charged substrates, e.g. the histones or the RPS6 *N*-terminal tail (^{129}VPRRLGPK**R**ASRIRKL^{144}). **c**, JMJD5 residues selected for cross-linking. We engineered a series of cysteine variants of residues (Q275C, Q277C, D280C, T306C, N308C, P324C and W414C) within ~12 Å radius of the metal at positions within the channel (**b**). We also substituted Cys residues at all positions on the RPS6 fragment peptide sequence (^{129}VPRRLGPK**R**ASRIRKL^{144}), except for the hydroxylated Arg residue. In addition, we substituted either 2 most nucleophilic (aa 217 and 232) or all 4 wildtype cysteines (aa 217, 232, 295 and 384) by alanines to avoid 'unwanted' disulfide formation with substrate-Cys variant. We then attempted crystallisation of various combinations of JMJD5-Cys variants (with 2-4 native cysteines substituted) in the presence of Mn(II)/ NOG substituting for Fe(II)/2OG and 16-mer RPS6-Cys (cysteines at different positions) or 5-mer RCCD1-wt (^{139}CA**R**AY^{143}) peptides. The cross-linking strategy enabled us to obtain crystal structures of JMJD5(Q275C).Mn.NOG.RPS6(A138C) (Complex 2), JMJD5(N308C).Mn.NOG.RCCD1 (Complex 3), JMJD5(N308C).Mn.2OG.RCCD1 (Complex 4) and JMJD5(W414C).Mn.NOG.RCCD1 (Complex 5).

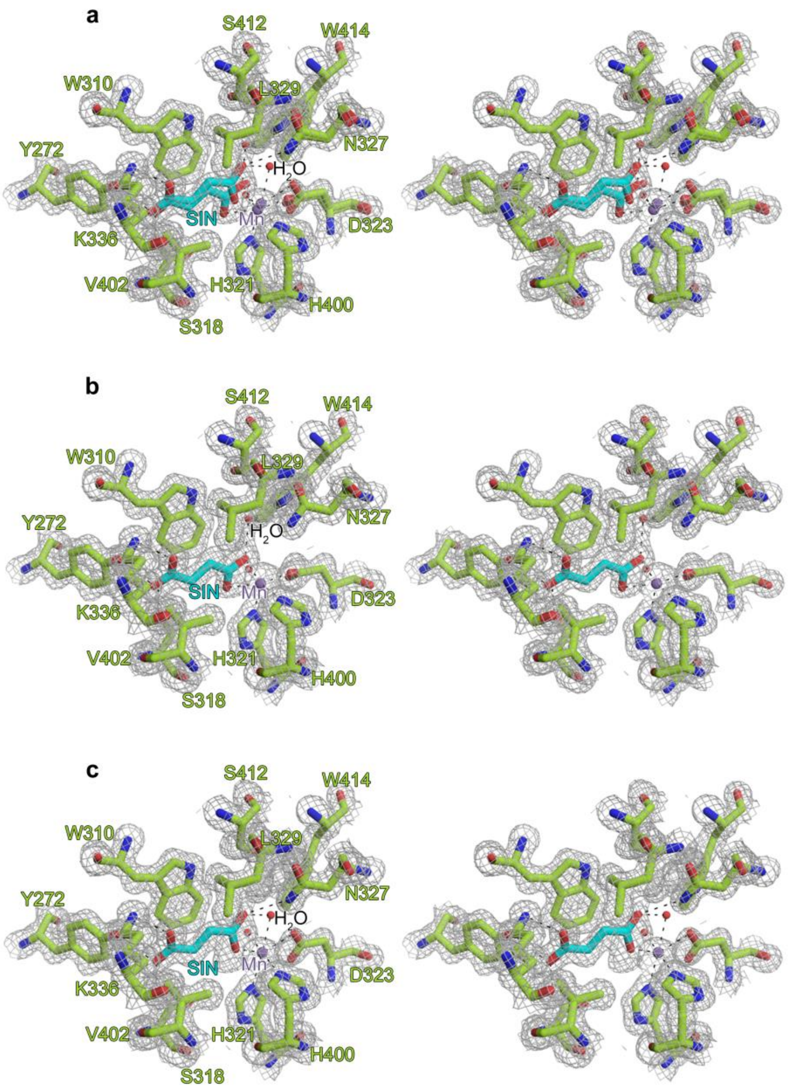

Supplementary Figure 11 | Stereoview representation of a JMJD5.succinate complex. **a**, JMJD5.Mn.succinate complex structure revealing a combination of major (~63%, **b**) and minor (~37%, **c**) forms, which show distinct bidentate (**b**) and monodentate (**c**) coordination of the metal (Mn, purple sphere) by succinate (cyan sticks). The structure reveals two orientations of the metal (violet sphere), metal-bound water (red sphere) along with conformational changes in the sidechains of DSBH residues, including those that are involved in metal/ 2OG/ succinate binding: D323 (βII), N327 (βIII), S412 (βVIII) and W414 (βVIII). Figure shows the $2F_o$-F_c map (grey meshes), contoured to 1.5σ.

Supplemental Tables

Supplementary Table 1 | List of RCCD1 peptides tested for hydroxylation by JMJD5. Corresponding sequences are mapped on to the RCCD1 sequence (RefSeq ID: NP_001017919). All peptides were synthesized as C-terminal amides.

MAEERPGAWFGFGFCG
GQELGSGRGRQVHSPS
VHSPSPLRAGVDICRV
LRAGVDICRVSASWSY
SYTAFVTRGGRLELSG
GSASGAAGRCKDAWASE
EGLLAVLRAGPGPEALL
VWAAESALRGEPLWAQN
AGEAQAGRLPLLPCARA
ARAYVSPRAPFYRPLAP
VSPRAPFYRPLAPELRA
PLAPELRARQLELGAEH
VFSWGGGRHGQLGHGT
TLEAELEPRLLEALQG
GQLALPTRNLAEDGET
AEDGETVAREATELNE
EDGSQVKRTGGAEDGA
AVKASCGSRHTAVVTR
RHTAVVTRTGELYTWG
DTTSLDRPRRVEYFVD
RLPLLPCARAYVSPRA
CARAYVSPRAPFYRPL
LPLLPCARAYVSPRAPF
LPLLPCAAAYVSPRAPF
LPLLPCARAYVSPAAPF

>NP_001017919.1 RCC1 domain-containing protein 1 [*Homo sapiens*]

MAEERPGAWFGFGFCGFGQELGSGRGRQVHSPSPLRAGVDICRVSASWSYTAFVTRGGRLELSGSASGAAGRCKDAWASEGLL AVLRAGPGPEALLQVWAAESALRGEPLWAQNVVPEAEGEDDPAGEAQAGRLPLLPCARAYVSPRAPFYR**PLAPELRARQLELGAE H**ALLLDAAGQVFSWGGGRHGQLGHGTLEAELEPRLLEALQGLVMAEVAAGGWHSVCVSETGDIYIWGWNESGQLALPTRNLAED GETVAREATELNEDGSQVKRTGGAEDGAPAPFIAVQPFPALLDLPMGSDAVKASCGSRHTAVVTRTGELYTWGWGKYGQLGHED TTSLDRPRRVEYFVDKQLQVKAVTCGPWNTYVYAVEKGKS

Supplementary Table 2 | Primer sequences used for cloning the JMJD5 variants.

Variants	Sequence
JMJD5 (183-416) Forward	5' TACTTCCAATCCACAGTCCCCCGGCTG 3'
JMJD5 (153-416) Forward	5' TACTTCCAATCCATGAGGCCTGCCCGTGG 3'
JMJD5 (S416) Reverse	5' TATCCACCTTTACTGCTACGACCACCAGAAGC 3'
C217A Forward	5' GAC CAC TGG CCG GCC ATG CAG AAG 3'
C217A Reverse	5' CTT CTG CAT GGC CGG CCA GTG GTC 3'
C232A Forward	5' GAG ATC GCT GGC GCA CGA ACT GTC CCA G 3'
C232A Reverse	5' CTG GGA CAG TTC GTG CGC CAG CGA TCT C 3'
C295A Forward	5' GC ATC CCC GAC TAC GCT AGC CTG 3'
C295A Reverse	5' CA GGC TAG CGT AGT CGG GGA TGC 3'
C384A Forward	5' GCC CCA TTC CTG TCC GCC ATC CTG TCT CCT G 3'
C384A Reverse	5' CAG GAG ACA GGA TGG CGG ACA GGA ATG GGG C 3'
E238A Forward	5' GTC CCA GTG GCA GTT GGT TCG AG 3'
E238A Reverse	5' CTC GAA CCA ACT GCC ACT GGG AC 3'
Y243A Forward	5' GTT GGT TCG AGG GCC ACA GAT GAG G 3'
Y243A Reverse	5' CCT CAT CTG TGG CCC TCG AAC CAA C 3'
L356A Forward	5' CAT GAC ACG CAC GCT CTC CAT AAC ACG AG 3'
L356A Reverse	5' CTC GTG TTA TGG AGA GCG TGC GTG TCA TG 3'
Q275A Forward	5' GGG TAC CTT GCT GCG CAC CAG CTC TTT G 3'
Q275A Reverse	5' CAA AGA GCT GGT GCG CAG CAA GGT ACC C 3'
W414A Forward	5' G GTC AGC TTC TGG GCG TCG TAG 3'
W414A Reverse	5' CTA CGA CGC CCA GAA GCT GAC C 3'

Supplementary Table 3 | Alanine variants of the RPS6 peptide ([129]VPRRLGPKRASRIRKL[144]) tested for hydroxylation by JMJD5 to identify the hydroxylated residue (a) and the predicted JMJD5 substrate sequences from human genome as revealed by bioinformatic analysis (b). Synthetic peptides (10 μM) were incubated with JMJD5 (1 μM) and co-substrates/ cofactors under the standard assay conditions (see Methods) for an hour at 37 °C and analysed by MALDI-MS. The extent of hydroxylation (%) was calculated from the relative intensities of peaks corresponding to the calculated *m/z* for unmodified and modified (+16) peptides. In addition to identifying the hydroxylated arginine, the results of Ala scan of $RPS6_{129-144}$ residues reveal sequence determinants for optimum arginyl hydroxylation (denoted by * in **b**), which were then utilised to predict other potential JMJD5 substrates/ interacting proteins in the human genome by a Pattern Hit Initiated (PHI)-BLAST search‡.

a

Peptide Sequence	% Hydroxylation
VPRRLGPKRASRIRKL	77
APRRLGPKRASRIRKL	86
VARRLGPKRASRIRKL	86
VPARLGPKRASRIRKL	87
VPRALGPKRASRIRKL	86
VPRRAGPKRASRIRKL	27
VPRRLAPKRASRIRKL	86
VPRRLGAKRASRIRKL	88
VPRRLGPARASRIRKL	87
VPRRLGPKAASRIRKL	0
VPRRLGPKRAARIRKL	22
VPRRLGPKRASAIRKL	79
VPRRLGPKRASRARKL	33
VPRRLGPKRASRIAKL	21
VPRRLGPKRASRIRAL	40
VPRRLGPKRASRIRKA	33

b

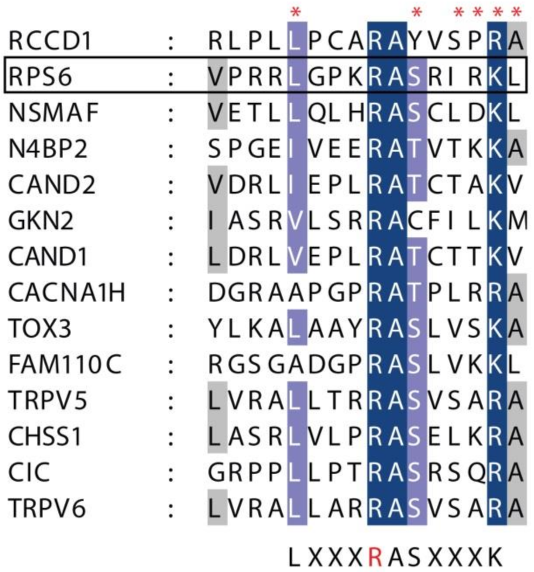

‡Parameters used for the PHI-BLAST search include: a PHI pattern syntax based on the sequence determinants (*) for arginyl hydroxylation - [LAVIM]XXX**R**A[STC]X[VILSTC]X[RK][LAVIM], where X is any amino acid; database - reference proteins (refseq_protein); scoring matrix - BLOSUM62 (for closely related sequences); E-value threshold - 1E-04.

Abbreviations used, NSMAF : Factor associated with neutral sphingomyelinase activation; N4BP2 : NEDD4 (E3 ubiquitin-protein ligase) -binding protein 2; CAND2 : Cullin-associated NEDD8-dissociated protein 2; GKN2 : Gastrokine-2; CAND1 : Cullin-associated NEDD8-dissociated protein 1; CACNA1H : Voltage-dependent T-type calcium channel subunit α-1H; TOX3 : TOX high mobility group box family member 3; FAM110C : Protein FAM110C; TRPV5 : Transient receptor potential cation channel subfamily V member 5; CHSS1 : Chondroitin sulfate synthase 1; CIC : Protein capicua homolog; TRPV6 : Transient receptor potential cation channel subfamily V member 6.

Supplementary Table 4 | Data collection and refinement statistics of JMJD5-apo/ ligand complexes.

	JMJD5-Apo	JMJD5.Mn(II). 2OG	JMJD5.Mn(II). Succinate
**PDB acquisition codes**	6F4M	6F4N	6F4O
**Data collection**			
No. of crystals	1	1	1
Data processing	HKL2000[8]	HKL2000[8]	HKL2000[8]
Space group	$P2_12_12_1$	$P3_221$	$P2_12_12_1$
Cell dimensions			
a, *b*, *c* (Å)	49.03, 66.44, 70.08	68.00, 68.00, 267.90	49.43, 64.41, 77.49
α, *β*, *γ* (°)	90, 90, 90	90, 90, 120	90, 90, 90
No. of molecules/ ASU	1	2	1
No. reflections	25416 (2481)*	24683 (2411)*	64327 (6378)*
Resolution (Å)	48.21 – 1.70 (1.76 – 1.70)*	49.16 – 2.54 (2.63 – 2.54)*	27.56 – 1.28 (1.33 – 1.28)*
R_{sym} or R_{merge}	0.101 (1.6)*	0.081 (1.1)*	0.081 (1.1)*
R_{pim}	0.040 (0.791)*	0.030 (0.423)*	0.035 (0.491)*
$I/\sigma I$	21.6 (1.9)*	25.1 (1.8)*	19.5 (1.9)*
CC (1/2)	0.998 (0.520)*	0.999 (0.738)*	0.996 (0.663)*
Completeness (%)	99.8 (99.7)*	99.9 (99.7)*	99.7 (100)*
Redundancy	12.6 (13.0)*	9.4 (8.7)*	7.1 (7.4)*
Wilson *B* value (Å^2)	24.8	68.6	16.0
**Refinement**	PHENIX[9]	PHENIX[9]	PHENIX[9]
Resolution (Å)	48.21 – 1.71	49.16 – 2.54	27.56 – 1.28
R_{work}/ R_{free}	0.170/ 0.187	0.199/ 0.230	0.146/ 0.174
No. atoms$^{\psi}$			
-Enzyme (A/B)	1972	1993/ 1996	2112
-Metal (A/B)	-	1/ 1	1
-Ligand (A/B)	-	10/ 10	8
-Water	207	189	312
B-factors$^{\psi}$			
-Enzyme (A/B)	34.7	82.3/ 86.2	23.0
-Metal (A/B)	-	65.4/ 63.5	14.1
-Ligand (A/B)	-	57.4/ 61.9	15.2
-Water	48.6	83.7	41.0
R.m.s deviations			
-Bond lengths (Å)	0.005	0.005	0.011
-Bond angles (º)	0.764	0.877	1.211

*Highest resolution shell shown in parentheses.

$^{\psi}$Polypeptide chain in parenthesis.

Supplementary Table 5 | Data collection and refinement statistics of JMJD5-substrate complexes.

	Complex 1	Complex 2	Complex 3	Complex 4	Complex 5
**PDB acquisition codes**	6F4P	6F4Q	6F4R	6F4S	6F4T
**Data collection**					
No. of crystals	1	1	1	1	1
Data processing	HKL2000[8]	HKL2000[8]	HKL2000[8]	HKL2000[8]	XDS[10], SCALA[11]
Space group	$P2_12_12_1$	$P2_12_12_1$	$P2_12_12_1$	$P2_12_12_1$	$P2_12_12_1$
Cell dimensions					
a, *b*, *c* (Å)	49.28, 64.93, 78.04	49.41, 65.07, 77.77	49.43, 64.81, 77.98	49.32, 65.61, 77.69	49.14, 65.17, 78.45
α, *β*, *γ* (°)	90, 90, 90	90, 90, 90	90, 90, 90	90, 90, 90	90, 90, 90
No. of molecules/ ASU	1	1	1	1	1
No. reflections	44727 (4347)*	95694 (9219)*	62115 (6098)*	44375 (2928)*	75316 (10919)*
Resolution (Å)	32.46 – 1.45 (1.50 – 1.45)*	49.91 – 1.12 (1.16 – 1.12)*	49.84 – 1.30 (1.35 – 1.30)*	33.43 – 1.46 (1.49 – 1.46)*	50.13 – 1.22 (1.29 – 1.22)*
R_{sym} or R_{merge}	0.090 (1.2)*	0.067 (1.1)*	0.079 (1.2)*	0.113 (1.3)*	0.049 (1.036)*
R_{pim}	0.035 (0.497)*	0.022 (0.411)*	0.031 (0.553)*	0.050 (0.602)*	0.023 (0.481)*
$I/\sigma I$	19.0 (1.8)*	32.9 (2.2)*	24.3 (1.7)*	17.0 (1.9)*	13.3 (1.8)*
CC (1/2)	0.997 (0.634)*	0.999 (0.707)*	0.998 (0.568)*	0.997 (0.602)*	0.999 (0.623)*
Completeness (%)	98.8 (97.7)*	98.7 (96.3)*	100 (100)*	99.9 (99.8)*	99.6 (99.9)*
Redundancy	8.8 (8.4)*	11.1 (9.9)*	9.0 (8.8)*	7.0 (7.0)*	5.6 (5.5)*
Wilson *B* value ($Å^2$)	17.5	12.8	15.6	12.7	16.0
**Refinement**	PHENIX[9]	PHENIX[9]	PHENIX[9]	PHENIX[9]	PHENIX[9]
Resolution (Å)	32.46 – 1.45	49.91 – 1.12	49.84 – 1.30	33.43 – 1.46	39.23 – 1.22
R_{work}/ R_{free}	0.146/ 0.168	0.139/ 0.153	0.143/ 0.162	0.136/ 0.152	0.141/ 0.154
No. atoms					
-Enzyme	2171	2188	2210	2282	2143
-Metal	1	1	1	1	1
-Ligand	10	10	10	10	10
-Substrate	22	17	27	39	28
-Water	337	373	372	354	353
B-factors					
-Enzyme	25.7	18.8	21.2	20.3	22.3
-Metal	12.8	9.9	11.5	9.9	13.3
-Ligand	14.6	10.2	13.2	12.1	13.6
-Substrate	38.8	40.6	28.5	24.0	31.2
-Water	47.5	35.7	43.8	41.4	41.4
R.m.s deviations					
-Bond lengths (Å)	0.009	0.010	0.016	0.008	0.018
-Bond angles (º)	1.005	1.162	1.401	0.962	1.511

*Highest resolution shell shown in parentheses.

Complex 1 : JMJD5.Mn.NOG.$RPS6_{129-144}$

Complex 2 : JMJD5(Q275C).Mn.NOG. $RPS6_{129-144}$(A138C)

Complex 3 : JMJD5(N308C).Mn.NOG.$RCCD1_{139-143}$

Complex 4 : JMJD5(N308C).Mn.2OG.$RCCD1_{139-143}$

Complex 5 : JMJD5(W414C).Mn.NOG.$RCCD1_{139-143}$

Supplementary References

1. Kato, M., *et al.* Crystal structure of a novel JmjC-domain-containing protein, TYW5, involved in tRNA modification. *Nucleic Acids Res.* **39**, 1576-1585 (2011).
2. Elkins, J. M., *et al.* Structure of factor-inhibiting hypoxia-inducible factor (HIF) reveals mechanism of oxidative modification of HIF-1α. *J. Biol. Chem.* **278**, 1802-1806 (2003).
3. Mantri, M., *et al.* Crystal Structure of the 2-Oxoglutarate- and Fe(II)-Dependent Lysyl Hydroxylase JMJD6. *J. Mol. Biol.*, (2010).
4. Chowdhury, R., *et al.* Structural basis for oxygen degradation domain selectivity of the HIF prolyl hydroxylases. *Nat. Commun.* **7**, 12673 (2016).
5. Chowdhury, R., *et al.* Ribosomal oxygenases are structurally conserved from prokaryotes to humans. *Nature* **510**, 422-426 (2014).
6. Yang, C. G., *et al.* Crystal structures of DNA/RNA repair enzymes AlkB and ABH2 bound to dsDNA. *Nature* **452**, 961-965 (2008).
7. Yi, C., *et al.* Iron-catalysed oxidation intermediates captured in a DNA repair dioxygenase. *Nature* **468**, 330-333 (2010).
8. Otwinowski, Z., Minor, W. & Jr., C. W. C. Processing of X-ray diffraction data collected in oscillation mode. In: *Methods Enzymol.* (ed^(eds). Academic Press (1997).
9. Adams, P. D., *et al.* PHENIX: a comprehensive Python-based system for macromolecular structure solution. *Acta Crystallogr. D Biol. Crystallogr.* **66**, 213-221 (2010).
10. Kabsch, W. Integration, scaling, space-group assignment and post-refinement. *Acta Crystallogr. D Biol. Crystallogr.* **66**, 133-144 (2010).
11. Winn, M. D., *et al.* Overview of the CCP4 suite and current developments. *Acta Crystallogr. D Biol. Crystallogr.* **67**, 235-242 (2011).
