# Supplementary material for: JMJD5 is a human arginyl C-3 hydroxylase
Source: Nat Commun. 2018 Mar 21;9:1180. doi: 10.1038/s41467-018-03410-w (PMC5862942; doi:10.1038/s41467-018-03410-w)
Supplement: Supplementary file 2 — Description of Additional Supplementary Files(PDF 170 kb) [file 41467_2018_3410_MOESM2_ESM.pdf]

Description of Supplementary Files

File Name: Supplementary Data 1
Description: **Ribosomal Peptide Library.** List of the amino acid sequences for synthetic peptides included in the ribosomal peptide library. Peptide sequences correspond to a subset of human ribosomal proteins as indicated (RefSeq IDs are listed below the protein name). In most cases, methionine residues have been substituted with valine or alanine residues to minimise non-specific oxidation of peptides during screening.
